# Cortical HFS-Induced Neo-Hebbian Local Plasticity Enhances Efferent Output Signal and Strengthens Afferent Input Connectivity

**DOI:** 10.1523/ENEURO.0045-24.2024

**Published:** 2025-02-03

**Authors:** Xiao Li, Xue Wang, Xiaohan Hu, Peng Tang, Congping Chen, Ling He, Mengying Chen, Stephen Temitayo Bello, Tao Chen, Xiaoyu Wang, Yin Ting Wong, Wenjian Sun, Xi Chen, Jianan Qu, Jufang He

**Affiliations:** ^1^Departments of Neuroscience, City University of Hong Kong, Kowloon, Hong Kong; ^2^Biomedical Science, City University of Hong Kong, Kowloon, Hong Kong; ^3^Research Centre for Treatments of Brain Disorders, City University of Hong Kong, Kowloon, Hong Kong; ^4^CAS Key Laboratory of Brain Connectome and Manipulation, the Brain Cognition and Brain Disease Institute, Shenzhen Institute of Advanced Technology, Chinese Academy of Sciences, Shenzhen 518055, China; ^5^Center of Regenerative Medicine and Health, Hong Kong Institute of Science and Innovation, Chinese Academy of Sciences, Shatin, Hong Kong; ^6^Department of Electronic and Computer Engineering, The Hong Kong University of Science and Technology, Kowloon, Hong Kong

**Keywords:** auditory cortex, cholecystokinin, high-frequency stimulation, interhemispheric cortical pathway, long-term potentiation, neo-Hebbian plasticity, recurrent excitation

## Abstract

High-frequency stimulation (HFS)-induced long–term potentiation (LTP) is generally regarded as a homosynaptic Hebbian-type LTP, where synaptic changes are thought to occur at the synapses that project from the stimulation site and terminate onto the neurons at the recording site. In this study, we first investigated HFS-induced LTP on urethane-anesthetized rats and found that cortical HFS enhances neural responses at the recording site through the strengthening of local connectivity with nearby neurons at the stimulation site rather than through synaptic strengthening at the recording site. This enhanced local connectivity at the stimulation site leads to increased output propagation, resulting in signal potentiation at the recording site. Additionally, we discovered that HFS can also nonspecifically strengthen distant afferent synapses at the HFS site, thereby expanding its impact beyond local neural connections. This form of plasticity exhibits a neo-Hebbian characteristic as it exclusively manifests in the presence of cholecystokinin release, induced by HFS. The cortical HFS-induced local LTP was further supported by a behavioral task, providing additional evidence. Our results unveil a previously overlooked mechanism underlying cortical plasticity: synaptic plasticity is more likely to occur around the soma site of strongly activated cortical neurons rather than solely at their projection terminals.

## Significance Statement

This manuscript reveals that cortical high-frequency stimulation (HFS) triggers the local release of cholecystokinin (CCK), a crucial neuromodulator for cortical plasticity, which is released at the HFS site from other cortical efferents rather than in a homosynaptic manner. Therefore, cortical HFS influences long-range cortical efferents through changes at the HFS location, not at the projection terminals. Additionally, the HFS-triggered locally released CCK strengthens long-range afferent synapses to the HFS site. This evidence suggests that a CCK-dependent neo-Hebbian mechanism underlies cortical plasticity.

## Introduction

Memory is believed to be stored in neural networks via changes in synaptic strength (Martin et al., 2000; Kandel, 2001). Long-term potentiation (LTP) and long-term depression are two forms of synaptic changes considered to be the basis of memory traces (Lømo, 1971; Bliss and Lømo, 1973; Artola and Singer, 1987; Bliss and Collingridge, 1993; Buonomano and Merzenich, 1998; Nabavi et al., 2014). High-frequency stimulation (HFS)-induced LTP is generally regarded as a homosynaptic Hebbian-type LTP (Lømo, 1971; Bliss and Lømo, 1973; Artola and Singer, 1987; Staubli and Lynch, 1987; Löwel and Singer, 1992; Buonomano and Merzenich, 1998; Hebb, 2005), with plasticity thought to occur at the recording site of which synapses receiving input from the stimulation site (Iriki et al., 1989; Kirkwood and Bear, 1994). LTP has been extensively studied in the hippocampus (Larkman and Jack, 1995). Unlike unidirectional feedforward projections in the hippocampus, most cortical connections are formed locally within neighboring excitatory neurons (Douglas et al., 1995; Douglas and Martin, 2004). However, previous cortical studies have largely concentrated on feedforward excitations, often neglecting excitatory feedback networks (Douglas et al., 1995). Cortical stimulation can generate positive feedback through local excitatory network, amplifying feedforward output signals via long-range efferent projections, a phenomenon known as recurrent excitation (Douglas et al., 1995). Thus, we hypothesize that HFS applied to induce cortical LTP may promote synaptic plasticity around the stimulation site. Consequently, the enhanced local cortical network leads to greater recurrent excitation and stronger stimulation outputs, thereby potentiating neural response recorded at the recording site. This is a local LTP that differs from the LTP of its efferent pathway. Notably, recent studies have shown difficulty in inducing homosynaptic LTP in visuoauditory cortical pathway or interhemispheric cortical pathway solely through optogenetic HFS of their cortical projections in a Hebbian manner (Li et al., 2023; Sun et al., 2024). Therefore, we deduce that HFS of the neocortex is more likely to enhance output signals around the stimulation site than the synaptic connectivity of its efferent terminals at the recording site. To differentiate these two components in the cortical LTP study, we placed electrodes in different hemispheres of the auditory cortex, ensuring no potential currents spread from the stimulation site to the recording site. In this study, we investigated HFS-induced LTP on urethane-anesthetized rats and demonstrated that cortical HFS enabled local LTP, which could enhance the output signal of interhemispheric propagation.

Cholecystokinin (CCK) is the most abundant neuropeptide in the brain (REHFELD, 1978; Moran and Schwartz, 1994). Extensive CCK-expressing neurons are found in the entorhinal cortex (Innis et al., 1979; Greenwood et al., 1981; Köhler and Chan-Palay, 1982), and the optogenetic HFS of entorhinoneocortical projections can trigger CCK release in the neocortex (Li et al., 2023). Previous studies have shown that CCK is essential for learning and memory (Nomoto et al., 1999; Hadjiivanova et al., 2003; Lo et al., 2008; Chen et al., 2019). In fact, exogenous or endogenous CCK can enable synaptic plasticity in different neural pathways alongside pre- and postsynaptic activity (Li et al., 2014; Chen et al., 2019; Zhang et al., 2020; Feng et al., 2021; Sun et al., 2024). Thus, CCK-modulated LTP can be seen as a neo-Hebbian process, with CCK serving as a third requirement supplementary to the two-factor (i.e., pre- and postsynaptic coactivation) classic Hebbian rules (Bailey et al., 2000; Lisman et al., 2011; Frémaux and Gerstner, 2015). In this study, we hypothesize that HFS in the neocortex could trigger CCK release, potentially inducing local neural plasticity. Given CCK's crucial role in facilitating LTP induction, we further reasoned that the presence of CCK may strengthen not only local connectivity but also other long-range afferents to this HFS site. Thus, we next applied HFS at the recording site to directly investigate the LTP induction of callosal afferent and to assess the physiological implications of neural responses to natural auditory stimuli. Finally, we demonstrated how HFS-induced neural plasticity modulates behavioral learning in rats using a two-alternative choice task, the results of which suggest that the plasticity induced by HFS occurs specifically at the HFS site. Our findings might uncover an overlooked mechanism underlying the formation of cortical plasticity.

## Materials and Methods

### Anatomical confirmation of the interhemispheric connectivity

Male Sprague Dawley rats (∼250 g) were housed in a standard light/dark cycle, with 12 h of light followed by 12 h of darkness. We anesthetized the animal with sodium pentobarbital (50 mg/kg, i.p.; Ceva Sante Animale). One-third of the initial dose was applied each hour throughout the surgery to maintain anesthesia. The body temperature was maintained between 37 and 38°C with a heating pad (RWD Life Science). Animals were mounted on a stereotaxic instrument (Narishige), and an incision was made in the scalp over the border between the parietal and temporal bones after the subcutaneous injection of a local anesthetic (lidocaine, 2%). A 1.5 × 2.5 mm craniotomy window was cut into the temporal bone 3.0–4.5 mm from the top edge (dorsal–ventral, DV) and bregma −3.0 to −5.5 mm (anterior–posterior, AP). We inserted an injector (nanoliter 2010, World Precision Instruments) perpendicularly from the cortical surface to the auditory cortex, using a motorized stereotaxic micromanipulator (Narishige). A glass pipette with a 20 µm tip diameter was fixed onto the nanoliter injector perpendicular to the surface of the temporal lobe. Cholera toxin B subunit (CT-B) conjugated to Alexa Fluor 488 (1 mg/ml, Molecular Probes) was injected into three locations with different coordinates (AP −3.5 mm, DV −3.8 mm; AP −4.5 mm, DV −3.8 mm; and AP −5.5 mm, DV −3.8 mm). At each location, the glass pipette tip was first advanced to −900 µm, and after 5 min of waiting, 300 nl CT-B was infused at a rate of 30 nl/min. The craniotomy window was filled with Kwik-Cast silicone gel (World Precision Instruments) before the incision was sutured. Then, erythromycin ointment was used on the external sutured skin to prevent wound infection. Animals were maintained on the heating pad until voluntary movements were observed and were then moved back to their cages. Five days later, animals were transcardially perfused with phosphate-buffered saline (PBS) and subsequently 4% paraformaldehyde (PFA).

### In vivo extracellular recordings in anesthetized rats

Anesthesia was induced by urethane sodium (2 g/kg, i.p.) and maintained throughout the surgery and during neuronal recordings, with periodic supplements. Atropine sulfate (0.05 mg/kg, s.c.) was administered 15 min before the induction of anesthesia to inhibit tracheal secretions. A local anesthetic (xylocaine, 2%) was liberally applied to the incision site. Animals were mounted in a stereotaxic device, and a midline incision was made in the scalp. A craniotomy was performed at each hemisphere (−2.5 mm to −6.5 mm posterior to the bregma and −2.0 mm to −4.5 mm ventral to sagittal suture for rats) to access the auditory cortex of each hemisphere, and the dura mater was opened. For all in vivo experiments, electrodes (<500 kΩ, FHC) were bilaterally and symmetrically inserted into Layer V of the auditory cortex. All electrodes could be used for either recording or stimulation. For all experiments involving drug infusions, the tips of glass pipettes were covered by 0.1 µl silicone oil to avoid leaking.

**Figure 1.**
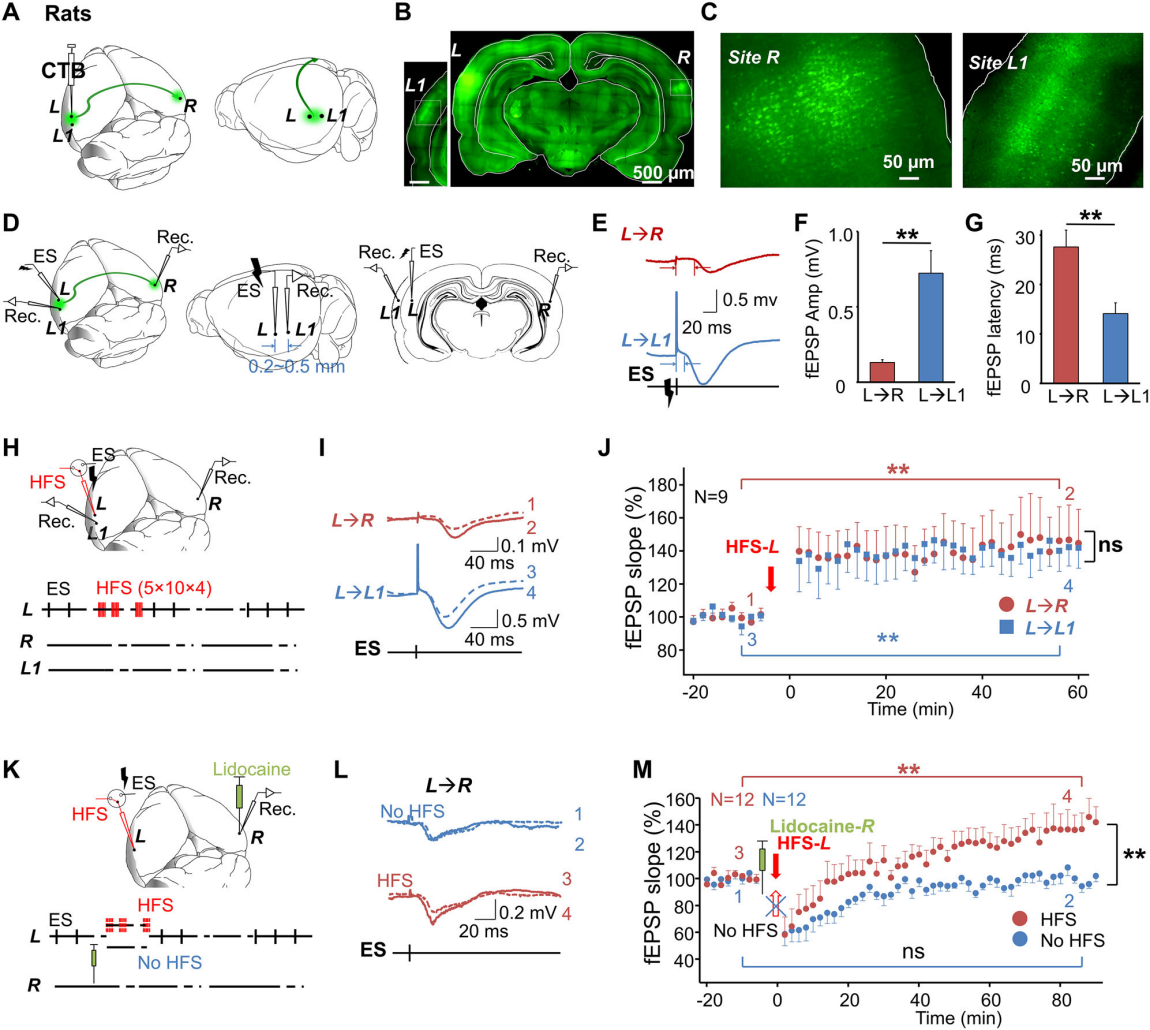
HFS induced stronger outputs from the stimulation site in the auditory cortex of rats. ***A***, CT-B (recombinant), Alexa Fluor 488 conjugate was injected into one hemisphere of the auditory cortex of rats. ***B***, Retrogradely labeled neurons were found in the contralateral auditory cortex (scale bar, 500 µm). ***C***, Left panel, Retrogradely labeled neurons in site ***R***. Right panel, Retrogradely labeled neurons in site ***L1*** (scale bars,50 µm). ***D***, Two electrodes were symmetrically implanted into the left (site ***L***) and right auditory cortices (site ***R***) to maximize their collateral connections. Site ***L1*** was adjacent to site ***L***. ***E***, Representative single fEPSP traces of the connections in ***L***→***R*** (top) and ***L***→***L1*** (bottom). ***F***, Bar charts showing the amplitudes of fEPSPs from the ***L***→***R*** and ***L***→***L1*** pathways. (***L***→***R*** vs ***L***→***L1***, 0.13 ± 0.02 mV vs 0.72 ± 0.15 mV; paired Student's *t* test; **p < 0.01; *N* = 9). ***G***, Bar charts showing the latencies of fEPSPs from the ***L***→***R*** and ***L***→***L1*** pathways. (***L***→***R*** vs ***L***→***L1***, 27.6 ± 3.4 ms vs 14.1 ± 2.2 ms; paired Student's *t* test; **p < 0.01; *N* = 9). ***H***, Top, Electrode placement. HFS was applied to site ***L***. Bottom, Experimental protocol. Probe ES and HFS were both applied to site ***L*** (ES-***L***, 0.1 Hz; HFS-***L***, 5-pulse burst at 100 Hz, 10 bursts at 5 Hz, repeated four times at 0.1 Hz). ***I***, Representative fEPSP traces of the connection ***L***→***R*** (1–2, at the end of each recording period) and ***L***→***L1*** (3–4, at the end of each recording period) before (dotted line) and after (solid line) HFS-***L***. ***J***, Normalized fEPSP slopes of the connections ***L***→***R*** (red circle) and ***L***→***L1*** (blue square) before and after HFS-***L***. Numbers 1–4 indicate the different time points of representative fEPSP traces (shown in Fig. 1*I*) selected during the recording sessions. (***L***→***R***, two-way RM ANOVA; **p < 0.01; ***L***→***L1***, two-way RM ANOVA; **p < 0.01; *N* = 9). Bars at the bottom indicate the periods over which the data are being pooled for the before and after measurements. ***K***, Electrode placement. The probe ES was applied to site ***L***; lidocaine was injected near the recording site ***R***; HFS was applied to site ***L***. ***L***, Representative fEPSP traces of pathway ***L***→***R*** before (dotted) and after (solid) lidocaine injection, followed by HFS (top, 1–2) or no HFS (bottom, 3–4). ***M***, Normalized fEPSP slopes before and after lidocaine injection at site ***R***, followed by HFS or no HFS (HFS group, red spot; two-way RM ANOVA; **p < 0.01; *N* = 12; no HFS group, blue spot, ns, two-way RM ANOVA; *p* = 0.78; *N* = 12). See Extended Data Figure 1-1 for more details.

In the experiments shown in Figure 1*H–J* and Figure 4*A–C*, two electrode arrays consisting of four electrodes each (three electrodes for recording or stimulation and one for reference) were inserted bilaterally and symmetrically into the auditory cortex. Electrode placements were confirmed by neuronal responses to stimulation applied to the contralateral hemisphere. Electrodes were positioned by a stepping-motor microdriver controlled from outside of the soundproofed chamber. One electrode was chosen from each side, and the locations of these electrodes were confirmed to receive mutual projections. One electrode was used for stimulation (site ***L***), and the other was used for recording (site ***R***). A second electrode was chosen from the stimulation site to act as the second recording electrode (site ***L1***). Recording electrodes were attached to a 16-channel head stage, preamplified, passed onto an acquisition processor, filtered with a bandwidth of 1–5,000 Hz, and stored using the TDT software [OpenEX, Tucker-Davis Technologies (TDT)]. We first recorded baseline fEPSPs in response to ES-***L*** (0.1 Hz at site ***L***) for 16 min from both recording electrodes (sites ***R*** and ***L1***). The maximum current recorded from the contralateral side (site ***R***) was defined as that which could elicit a saturated fEPSP. To induce cortical LTP, we determined the necessary ES-***L*** current to evoke the maximum fEPSP at site ***R*** and applied 40% of that as the testing current and 80% of that as the HFS current (Extended Data Fig. 1-1*A*). The protocol of HFS was illustrated in Extended Data Figure 1-1*B* (each burst included five 0.5 ms pulses at 100 Hz, and each block consisted of 10 bursts at 5 Hz, for a total of four blocks with an intertrial interval of 10 s). Extended Data Figure 1-1*C* shows an example of fEPSP evoked by HFS. fEPSPs were then recorded from both sites for 16 min before and 60 min after the HFS, which was applied to the stimulation site ***L*** (HFS-***L***) for the experiment shown in Figure 1*J* and at the recording site ***R*** (HFS-***R***) in the experiment shown in Figure 4*C*.

In the experiment shown in Figure 1*K–M*, only one recording electrode was placed on the hemisphere (site ***R***) contralateral to the ES site (site ***L***), and a glass pipette was inserted adjacent to the recording site ***R*** for drug infusion. After recording baseline fEPSPs in response to ES-***L*** for 16 min, we infused lidocaine (2 mg/ml, 0.7 µl in saline) at a rate of 0.07 µl/min. HFS-***L*** was then delivered to the ES site (site ***L***). No HFS was provided in the control group. The fEPSP recording was resumed for 90 min after the neurons at site ***R*** started responding to ES-***L*** again. The reappearance of spontaneous neuronal activity served as an indicator of a clear neuronal response before the recording was resumed.

In the experiment shown in Figures 3*A* and 4*D*, a glass pipette was inserted adjacent to the stimulating electrode (site ***L***; Fig. 3*A*) or recording electrode (site ***R***; Fig. 4*D*) to infuse CCK-8s (0.5 µl, 10 ng/µl, Tocris Bioscience) or artificial cerebrospinal fluid (ACSF) into the auditory cortex, at a rate of 0.1 µl/min. ES-***L*** (0.1 Hz) was continuously presented during the infusion. fEPSPs in response to ES-***L*** were recorded for 16 min before and 60 min after the infusion.

In the experiment shown in Figure 4*D*, a glass pipette was inserted adjacent to the recording electrode (site ***R***) to infuse CCK-8s (0.5 µl, 10 ng/µl, Tocris Bioscience) or ACSF into the auditory cortex of rats at a rate of 0.1 µl/min. ES-***L*** (0.1 Hz) was continuously presented during the infusion. fEPSPs in response to ES-***L*** were recorded for 16 min before and 60 min after the infusion.

In the experiment shown in Figure 3*B*, a glass pipette was inserted adjacent to both the recording electrodes (site ***R***) and the stimulating electrode (site ***L***). fEPSPs were recorded for 16 min before the infusion of L365,260 (10 µg/ml, 0.5 µl in 95% ACSF/5% DMSO, Tocris Bioscience) at a rate of 0.1 µl/min at site ***L,*** where HFS-***L*** was subsequently applied. fEPSPs in response to ES-***L*** were recorded for 60 min. Then, another infusion was delivered to the recording site ***R***, followed by HFS-***L*** again. fEPSP in response to ES-***L*** was recorded for another 60 min.

Similarly, in the experiment shown in Figure 4*E*, fEPSPs were recorded for 16 min before the infusion at the recording site ***R***, where HFS-***R*** was subsequently applied. fEPSPs in response to ES-***L*** were recorded for 60 min. Then, another infusion was delivered to the stimulation site ***L***, followed by HFS-***R*** at the recording site ***R***. fEPSP in response to ES-***L*** was recorded for another 60 min.

In the experiment shown in Extended Data Figure 4-1, auditory stimuli were digitally generated using a computer-controlled TDT workstation and delivered through a speaker. Electrodes were inserted into both hemispheres of the auditory cortex. Correct electrode placements were confirmed by neuronal responses to auditory stimuli. Baseline fEPSPs were recorded in response to noise for 16 min before HFS was delivered through the electrode at site ***R***. fEPSPs were then recorded for 60 min.

The data for the before and after measurements in the experiment are derived from the last 10 min of the baseline recording and the last 10 min of the post-HFS/injection recording. Mean fEPSP slopes were calculated by linear regression and compared using one-way or two-way analysis of variance (ANOVA) with repeated measures (RM) followed by Bonferroni-adjusted pairwise comparison.

### Two-photon calcium imaging of neuronal responses after HFS

Chronic window implantation: Thy1-GCaMP6 s transgenic mice (Jackson Laboratory #025776), 6–8 weeks old, were given subcutaneous injections of dexamethasone (2 mg/kg) and carprofen (5 mg/kg) at least 2 h before surgery to prevent inflammation and edema. Mice were deeply anesthetized using pentobarbital (10 mg/kg, i.p.) and given an intramuscular injection of atropine (0.05 mg/kg). Body temperature was maintained at 38°C using a feedback-controlled heating blanket. The scalp fur was trimmed, the scalp was disinfected with 70% ethanol swabs, and a patch of skin was removed. The temporal muscle was detached from the skull and removed, and the skull was cleaned and dried. A square craniotomy (3 mm) was made in the center of the auditory cortex, and the bone patch was removed. After cleaning the debris and the skull edges, a square coverslip (with a 0.5-mm-diameter hole) was placed above the auditory cortex. Instant glue was used to seal the edge, followed by the application of one layer of C&B Metabond, and dental cement was used to fix the head-plate. Kwik-Cast was used to protect the coverslip.

Two-photon calcium imaging: The imaging experiments were conducted using a homemade, upright, two-photon microscopy (Chen et al., 2018). Briefly, the microscope consists of a tunable femtosecond laser (Chameleon, Ultra II, Coherent), a dual-axis galvanometric scanner (6210H, Cambridge Technology), and a water-immersion objective (XLUMPLFLN 20XW, Olympus), with a 2 mm working distance. A multialkali photomultiplier tube (H11461-03, Hamamatsu Photonics), combined with a low-noise current amplifier (SR570, Stanford Research), was used for fluorescence detection. Microscope control and image acquisition were performed using a multifunctional data acquisition device (PCIe-6361, National Instruments), operated by an integrated, custom-written, C# program. Prior to the experiments, the mice were sedated with chlorprothixene (0.5 mg/kg, i.m.) and anesthetized with isoflurane (5%). The mice were then lightly anesthetized (0.5% isoflurane), with their heads fixed on a custom stage, and placed under the microscope using a warming blanket (38°C) to maintain stable body temperatures. For calcium imaging, images (256 × 200 pixels, 1 ms per line, the field of view of 250 × 200 µm) were acquired at 5 Hz for 2 min per trial. For electrophysiological stimulation, a tungsten microelectrode (Lot #849281, 100 KΩ FHC) clamped on a micromanipulator (model MM-3, Narishige) was inserted into the auditory cortex (500 µm depth) via a cranial window access hole. The stimulation current was powered by a stimulus isolator (ISO-Flex, AMPI), with an input TTL signal generated by an Arduino board. ES of 0.5 ms duration was delivered at 0.1 Hz. ES started at 10 µA during the first trial and was increased using 15 µA increments until 160 µA was reached. After eight trials of the baseline, HFS (five pulses at 100 Hz for each burst, 10 bursts at 5 Hz for each train, using four trains, with an intertrain interval of 10 s) was delivered. Neuronal responses to single-pulse ES were further recorded for 20 trials after HFS.

Data analysis: We adopted the *tif* file format for use in the MATLAB program. The file was registered using the TurboReg plugin in ImageJ. The region of interest was manually selected, and the MATLAB script was used to generate the dF/*F* curve based on the raw data (Jia et al., 2011).

#### fEPSPs evoked by electrical stimulation or laser stimulation at the projecting terminals with opsin expression in mice

Virus injection: Animals were anesthetized with pentobarbital (100 mg/kg) and supplemented with atropine (0.05 mg/kg, Sigma-Aldrich). The anesthetized mice were head-fixed on a stereotaxic device (RWD Life Science).

In the experiments shown in Figure 4*F–H*, C57BL/6 wild-type (C57) mice were adopted as the subjects. pAAV-Syn-ChrimsonR-tdTomato (300 nl, 5.2E12 gc/ml, Addgene) virus was injected into one hemisphere of the auditory cortex (AP, −2.7 mm posterior to the bregma; ML, −4.2 mm lateral to the midline; DV, 0.7 mm below the pia), at a rate of 30 nl/min (Nanoliter Injector, World Precision Instruments).

Optogenetics in vivo fEPSP recordings: A craniotomy was performed (−2 mm to −4 mm posterior and −1.5 mm to −3 mm ventral to sagittal suture) to access the auditory cortex of each hemisphere, and the dura mater was opened. Electrodes were bilaterally inserted into Layer V of the auditory cortex where the virus was injected and its symmetric site. Glass pipette electrodes rather than metal electrodes were used at the recording site ***R*** to avoid the photoelectric effect, and an optical fiber was lowered onto the surface of recording site ***R*** for the laser stimulation. During the experiment, laser (LS-***R***, ≈5 mW, 5 ms duration) and ES-***L*** were alternately delivered with an interval of 5 s. fEPSPs in response to ES-***L*** and LS-***R*** were recorded 16 before and 60 min after the application of HFS***-L***; HFS was then delivered to site ***R*** (HFS-***R***), and fEPSPs were recorded for another 60 min.

Mean fEPSP slopes before and after the pairings were normalized, calculated by linear regression, and analyzed using two-way ANOVA followed by Bonferroni’s pairwise comparison.

### Fiber photometry

In all the fiber photometry experiments, pAAV-hSyn-CCK1.0 (3.5E13 gc/ml, Vigene Biosciences) was injected into the auditory cortex of C57 or CCK-KO [CCK-CreER, Cck^−/−^, Ccktm2.1(Cre/ERT2)Zjh/J] mice (AP, −2.6 and −3.2 mm posterior to the bregma; ML, −4.2 mm lateral to the midline; DV, −0.90 mm below the pia). This G-protein–coupled receptor (GPCR) activation-based CCK sensor, GRABCCK, was developed by inserting a circular-permutated green fluorescent protein (cpEGFP) into the intracellular domain of CCK-B receptor (CCKBR; Jing et al., 2019). When the endogenous or exogenous ligand (CCK) binds to the CCKBR, a conformational change in cpEGFP will happen as well as an increase in fluorescence intensity, and the CCK activity could be visualized in vivo. After a 6 week recovery, a craniotomy was performed to access the auditory cortex (−2 mm to −4 mm posterior and −1.5 mm to −3 mm ventral to sagittal suture), and the dura mater was opened. An optic fiber (400 µm diameter, 0.22 NA, Thorlabs) was lowered into the auditory cortex (100–200 µm from the brain surface) to record the signal of the CCK sensor (Fig. 3*C–E*). Before signal recording, the optic fiber was lowered to the brain surface at different sites to confirm the best site for the recording, where we were able to capture the strongest fluorescence signal of the CCK sensor. This optic fiber cannula was attached to a single fluorescent MiniCube (Doric Lenses) with built-in dichroic mirrors and LED light sources through a fiber patch cord. The excitation light at 470 and 405 nm were released by two fiber-coupled LEDs (M470F3 and M405FP1, Thorlabs) and were sinusoidally modulated at 210 and 330 Hz, respectively. The 473 nm channel is the GRAB_CCK_ channel, and the 405 nm channel is used as the isosbestic control channel. An LED driver (LEDD1B, Thorlabs) coupled to the RZ5D processor (TDT) managed the excitation light's intensity through the software Synapse. The emission fluorescence was captured and transmitted by a bandpass filter in the MiniCube. To avoid photobleaching, the excitation light intensity at the tip of the patch cord's tip was adjusted to <30 µW. The fluorescent signal was then detected, amplified, and transformed into an analog signal by the photoreceiver (Doric Lenses). The analog signal was then digitalized by the RZ5D processor and subjected to a 1 kHz low-pass analysis using the Synapse software. For the electric stimulation experiments, a tungsten-stimulating electrode with low impedance (<100 kΩ, FHC) was lowered into the auditory cortex site ***R*** (400–700 µm from the brain surface) with the recording optic fiber, another stimulating electrode was placed symmetrically into the left auditory cortex (site ***L***). The depth of the electrode and the fiber were controlled by a stepping-motor microdriver outside the chamber. The fluorescent signal was recorded 20 s before and 45 s after the HFS (50–150 µA) application (of either site ***R*** or site ***L***).

#### Fiber photometry analysis

Analysis of the signal was done by the custom-written MATLAB (MathWorks) codes. We first extracted the signal of 473 and 405 nm channels corresponding to the defined period before and after each stimulus. A fitted 405 nm signal was created by regressing the 405 nm channel onto a linear fit of its respective 473 channel (MATLAB *polyfit* function). The fluorescence change (Δ*F*/*F*) was then calculated with the formula (473 nm signal − fitted 405 nm signal) / fitted 405 nm signal (Bruno et al., 2021).

### Two-alternative choice task behavioral experiment

For the experiments shown in Figure 5, rats were anesthetized with sodium pentobarbital (50 mg/kg, i.p.), and anesthesia was maintained at a dose of 15 mg/kg/h. Atropine sulfate (0.05 mg/kg, s.c.) was administered 10 min before anesthesia to inhibit tracheal secretions. A local anesthetic (xylocaine, 2%) was liberally applied to the incision site. The surgical procedure was the same as that described above.

After the surgery, we implanted homemade electrode arrays, consisting of five electrodes (four electrodes for stimulation or recording and one as the reference electrode), symmetrically into both hemispheres of the auditory cortex. All electrodes were made of insulated tungsten wire (California Fine Wire or A-M Systems), with an impedance of 100 KΩ. Ground electrodes were connected to screws on the skull. The electrode array was held by a micromanipulator and inserted into the cortex. Electrodes in both hemispheres were placed symmetrically, to a depth of 800–1,000 µm. ES application through the left hemisphere was to induce neural responses near the electrodes in the contralateral hemisphere and vice versa. The skull opening was covered by a layer of silicone (World Precision Instruments). The connection sockets for the electrodes were cemented to the skull. Rats were then housed in their home cages for recovery.

Five days after the surgery, the rats underwent behavioral training, during which they learned to retrieve a water reward. Water was restricted to 30% of their regular intake prior to training. The rats were placed in a homemade cage with three horizontally aligned holes and were manually guided to poke their noses into the center hole before moving to the left or right side hole, where a drop of water (15–25 µl) was delivered. Infrared sensors in the holes detected the arrivals of the rats. The rats then underwent formal training consisting of four stages.

During Stage 1, rats could obtain the water reward from either the left or right side holes after poking their noses into the center hole. During Stage 2, a nose-poking in the center hole triggered ES-***L*** or ES-***R*** (five pulses at 100–200 µA in 5 Hz), which indicated a specific hole for reward availability. Pulses were generated by the TDT system and delivered through the electrodes via isolators (ISO-Flex, A.M.P.I). Stimulation of the left hemisphere indicated reward availability in the rightmost hole, while stimulation of the right hemisphere indicated reward availability in the leftmost hole. A drop of water was delivered only when infrared sensors detected the animal's arrival at the correct hole within 2 s from the onset of stimulation. Electrical stimulations were delivered to one hemisphere in one session (10 trials) and the opposite hemisphere in the next session until an 85% success rate was achieved. During Stage 3, stimulations were delivered to alternating hemispheres, for five trials each, until a success rate of 85% or higher was achieved. During Stage 4, electric stimulations were delivered to either hemisphere in a pseudorandom manner. The training finished when the rat reached a success rate of 85% or higher, and the number of pulses during each stimulation was gradually reduced from 5 to 1. We changed the current of the ES cue to adjust the animal's performance. A weaker stimulating current resulted in lower success rates. The stimulation current that resulted in a success rate of ∼80% was chosen as the stimulation current for the following experiment, and the rat then underwent a baseline test with the selected current of stimulation. During the task, if the rat made the wrong choice in response to the ES cue, the center hole would stop detecting for 3 s as a punishment.

In the experiment of Figure 5*E*, we increased the current of the ES cue by ∼20% for one hemisphere and maintained the current of the ES cue to the other hemisphere at baseline levels, following baseline testing in response to ES cues to either hemisphere. Task performance was reexamined after changing the current of the ES cue.

The rat was then anesthetized with sodium pentobarbital. Neuronal responses (fEPSPs) to ES applied to the contralateral hemisphere were measured before HFS. HFS (paradigm, Extended Data Fig. 5-1*H*) was applied three times at 30 min intervals, and fEPSPs in response to ES applied to the contralateral hemisphere were recorded for 30 min after each HFS session (Extended Data Fig. 5-1*C*–*G*). Rats' performances on the task were examined the day after the above manipulation.

### Immunohistology and image acquisition

Mice were anesthetized by an overdose of pentobarbital sodium and transcardially perfused with 30 ml cold PBS and 30 ml 4% (*w*/*v*) PFA. The brain tissue was removed, postfixed with 4% PFA, and treated with 30% (*w*/*v*) sucrose in 4% PFA at 4°C for 2–3 d. The brain tissue of 30 µm thickness was sectioned on a cryostat (Leica CM3500) and preserved with antifreeze buffer [20% (*v*/*v*) glycerol and 30% (*v*/*v*) ethylene glycol diluted in PBS] at −20°C.

Brain slices were washed three times per 10 min each time before blocking with the buffer [10% (*v*/*v*) goat serum in PBS with 0.2% (*v*/*v*) Triton X-100] at room temperature. After 2 h of blocking, the slices were bathed in the rabbit anti-CamKll (Abcam, ab5683, 1:1,000, shown in Extended Data Fig. 2-1*A*), mouse -anti-GAD67 (Millipore Sigma, MAB5406, 1:500, shown in Extended Data Fig. 2-1*C*), and goat anti-CCKBR (Abcam, ab77077, 1:1,000, shown in Extended Data Fig. 2-1*E*) which was prepared with blocking buffer shaking for 36 h at 4°C. After washing four times in PBS (each time for 10 min), sections were incubated with the corresponding fluorophore-conjugated secondary antibodies (1:250): donkey anti-rabbit 647 (Jackson Immunostar 711-605-152), donkey anti-goat 647 (Jackson Immunostar 705-605-147), or donkey anti-mouse 594 (Jackson Immunostar 715-585-150) for 2.5 h at 25°C. Sections were then rinsed with PBS three times before DAPI staining [1:5,000 (*v*/*v*) diluted in PBS] and mounting.

All the sections were mounted with 70% (*v*/*v*) glycerol in PBS on slides. Image acquisition (10×, 20×, 63×, and 100× magnification) was performed with a Nikon A1HD25 confocal microscope (Nikon).

### IHC images analysis

The images were analyzed with NIS element (Nikon) or ImageJ (NIH). For counting immunoreactive signals, the 8 bit grayscale image was background subtracted before applying a threshold to all images. The threshold was adjusted within 10% of the average intensity, and signals at or above the threshold are considered immunopositive.

For quantification of GCaMP6s^+^ neurons coexpressing CamKll (Extended Data Fig. 2-1*A*), 20× images or 100× **Z**-stack images were acquired for 2–3 fields of view surrounding the AC of each slice. The percentage of GCaMP6s^+^ cells, as identified by their fluorescent protein expression, overlapping with the CamKll was calculated for each field of view. The neurons were counted blindly by others using the NIS element and ImageJ.

### Quantification and statistical analysis

All statistical analyses (including paired *T* test, one-way RM ANOVA, two-way RM ANOVA) were done in SPSS (IBM). Pairwise comparisons were adjusted by Bonferroni’s correction. *N* represents the number of animals, and *n* represents the number of recordings (change to a different stimulation/recording site). Statistical significance was set at *p < 0.05 and **p < 0.01 (Table 1).

**Table 1.** Statistical table

	Data structure	Type of test	Power
Figure 1*F*	Normal distribution	Paired Student's *t* test	*p* < 0.01
Figure 1*G*	Normal distribution	Paired Student's *t* test	*p* < 0.01
Figure 1*J*	Normal distribution	Two-way RM ANOVA	***L*** to ***R***: before vs. after HFS-***L***, *p* < 0.001; ***L*** to ***L1***: before vs. after HFS-***L***, *p* < 0.01
Figure 1*M*	Normal distribution	Two-way RM ANOVA	Control group, *p* = 0.858; lidocaine group, *p* < 0.01
Figure 2*F*	Normal distribution	Paired Student's *t* test	*p* < 0.01
Figure 2*G*	Normal distribution	Paired Student's *t* test	*p* < 0.05
Figure 2*H*	Normal distribution	Two-sample Kolmogorov– Smirnov test	*p* < 0.01
Figure 3*A*	Normal distribution	Two-way RM ANOVA	Before vs after CCK-***L***, *p* < 0.01; before vs after ACSF-***L***, *p* = 0.705
Figure 3*B*	Normal distribution	One-way RM ANOVA	Before vs after L365-***L*** and HFS-***L***, *p* = 0.353; before vs after L365,260-***R*** and HFS-***L***, *p* < 0.01
Figure 3*E*	Normal distribution	Two-way ANOVA	Before vs after HFS-***R*** in C57, *p* < 0.01; before vs after HFS-***L*** in C57, *p* = 1.0; before vs after HFS-***R*** in CCK-KO, *p* = 1.0; after HFS-***R*** in C57 vs CCK-KO, *p* < 0.01; after HFS-***R*** vs HFS-***L*** in C57, *p* < 0.01
Figure 4*C*	Normal distribution	Two-way RM ANOVA	Before vs after HFS-***R***, *p* < 0.01; before vs after HFS-***R***, *p* = 0.95; *N* = 9 rats; ***L*** to ***R*** vs ***L*** to ***L***1 after HFS-R, *p* < 0.01
Figure 4*D*	Normal distribution	Two-way RM ANOVA	Before vs after CCK infusion, *p* < 0.01; before vs after ACSF infusion, *p* = 0.820
Figure 4*E*	Normal distribution	One-way RM ANOVA	Before vs after L365-***R*** and HFS-***R***, *p* = 1.0; before vs after L365-***L*** and HFS-***R***, *p* < 0.01
Extended Data Figure 4-1*C*	Normal distribution	Two-way RM ANOVA	Site ***L*** recording, before vs after HFS, *p* = 0.520; site ***R*** recording, before vs after HFS, *p* < 0.01
Figure 4*H*	Normal distribution	Two-way RM ANOVA	Response to ES after HFS-***L*** *p* < 0.01; response to LS-***R*** after HFS-***L***, *p* = 1.0; response to ES after HFS-***R***, *p* < 0.01; response to LS-***R*** after HFS-***R***, *p* < 0.01
Figure 5*E*	Normal distribution	Paired Student's *t* test	Response to the increased ES-***L***, *p* < 0.01; response to the unchanged ES-***R***, *p* = 0.046
Figure 5*H*, left	Normal distribution	Paired Student's *t* test	Response to ES-***L***, *p* = 0.011
Figure 5*H*, right	Normal distribution	Paired Student's *t* test	Response to ES-***R***, *p* < 0.01

### The code accessibility statement

The MATLAB scripts and functions utilized for data processing in this study are available upon request. Researchers interested in accessing the code for replication or further investigation may contact the corresponding author directly for assistance.

10.1523/ENEURO.0045-24.2024.d1 Extended Data 1 This MATLAB script is designed to process and analyze the stimulus-induced neural responses in the form of fEPSPs recorded during electrophysiological experiments. It calculates the slope or amplitude of these fEPSPs to quantify synaptic strength and response dynamics. Download Extended Data 1, ZIP file.

## Results

### HFS induces stronger outputs from the stimulation site

Cortical neurons receive inputs from neighboring excitatory neurons, and recurrent excitation during electrical stimulation (ES) might occur due to this local neural connectivity (Douglas et al., 1995). We hypothesized that HFS at the neocortex could enable neuroplasticity around the HFS site. This enhanced local neural connectivity at the stimulation site would intensify recurrent excitation and subsequently facilitate stronger outputs generated by ES after HFS compared with before HFS. To test this hypothesis, we selected different hemispheres as the stimulation site ***L*** and the recording site ***R***, respectively, and investigated the induction of LTP in the interhemispheric pathway ***L***→***R*** in rats. The two sites were positioned far apart to avoid direct activation of neurons in the recording site caused by the ES at the stimulation site. We first confirmed the existence of commissural projections between the two sites by injecting CT-B conjugated to Alexa Fluor 488 into one hemisphere (Fig. 1*A*) and observing retrogradely labeled neurons in the contralateral hemisphere (Fig. 1*B*,*C*). This confirmed that neurons in the auditory cortex project to the symmetric location in the contralateral hemisphere of the rat brain. Local cortical connectivity around the CT-B injection site ***L*** was evidenced by the labeled neurons adjacent to site ***L*** (Fig. 1*A–C*, site ***L1***).

10.1523/ENEURO.0045-24.2024.f1-1 Figure 1-1 **HFS induced stronger outputs from the stimulation site**. (**A**) Representative relationship between input currents ES-***L*** and evoked fEPSPs and site ***L1*** and site ***R***. V_ES_: 40% of the current that could evoke the maximum fEPSP at site ***R*** was used for testing; V_HFS_: 80% of the current that could evoke the maximum fEPSP at site ***R*** was used for HFS. (**B**) HFS protocol. Each burst includes five 0.5-ms pulses at 100  Hz, and each block consists of 10 bursts at 5  Hz, for a total of 4 blocks with an inter-block interval of 10  s. (**C**) The fEPSP was evoked by HFS (***L***→***L1***). (**D**) Example of neural activities after lidocaine infusion. Top: Neural response was silenced 1 minute after lidocaine infusion. Middle: Neural response was silenced 15  min after lidocaine infusion. Bottom: Neural response was fully recovered 30  min after the lidocaine infusion in this example. Download Figure 1-1, TIF file.

During the electrophysiology experiment, rats were anesthetized with urethane. We inserted two electrodes into site ***L*** (marked as site ***L*** and site ***L1***) and one electrode at the symmetrical site ***R*** in the contralateral hemisphere (Fig. 1*D*). Low-impedance electrodes were used to deliver stimulation currents and record neural responses in the form of local field excitatory postsynaptic potentials (fEPSPs). We first applied ES to site ***L*** (ES-***L***) and recorded the fEPSPs from both the contralateral site ***R*** and the nearby site ***L1*** (Fig. 1*D*; with example traces shown in Fig. 1*E*). The average amplitude of the fEPSPs at site ***R*** was smaller than that at site ***L1*** (Fig. 1*F*; 0.13 ± 0.02 mV vs 0.72 ± 0.15 mV; *p* < 0.01; paired Student's *t* test), indicating stronger local connectivity (***L***→***L1***) compared with interhemispheric connectivity (***L***→***R***). The average latency of the fEPSPs recorded at site ***R*** was also longer than those at site ***L1*** (Fig. 1*G*; 27.6 ± 3.4 ms vs 14.1 ± 2.2 ms; *p* < 0.01; paired Student's *t* test). Following baseline probe testing (Fig. 1*H*, ES-***L***), HFS current was delivered to the electrode at site ***L*** (HFS-***L***). The fEPSPs in response to ES-***L*** were potentiated at both sites ***R*** (***L***→***R)*** and ***L1*** (***L***→***L1)*** after HFS-***L*** (Fig. 1*I*,*J*; RM ANOVA; significant interaction *F*_(1,88)_ = 0.021; *p* = 0.885; ***L***→***R***, 100.6 ± 1.6% vs 144.9 ± 8.7%, before vs after HFS-***L***, pairwise comparison; *p* < 0.01; ***L***→***L1***, 99.1 ± 1.5% vs 141.9 ± 5.1%, before vs after HFS-***L***, pairwise comparison; *p* < 0.01; *N* = 9 rats).

Although we demonstrated that the local LTP (***L***→***L1***) exists after cortical HFS, this alone does not confirm whether the local LTP, resulting in stronger outputs, accounts for the potentiation recorded at site ***R***. Additionally, the current understanding appears contradictory to our hypothesis: the LTP recorded at site ***R*** should be homosynaptic LTP of ***L***→***R*** pathway, with plasticity occurring at the projection terminals in the recording site ***R***. To investigate this, we inactivated the neurons at site ***R*** while applying HFS to site ***L*** (HFS-***L***) to examine whether the LTP of ***L***→***R*** could be blocked, under the assumption that no neuroplasticity could occur if the targeted neurons are inactivated (Wiesel, 1982; Welsh and Harvey, 1991; Nordholm et al., 1993). We infused lidocaine (2.0%, 0.7 µl) into site ***R*** (Fig. 1*K*) of rats after baseline recording. Lidocaine temporarily suppressed neuronal activity at site ***R*** (Extended Data Fig. 1-1*D*). We resumed the fEPSP recording after the neurons at site ***R*** began responding to ES-***L*** again. In the control group, no HFS was applied after the lidocaine infusion. The fEPSP slope remained unchanged after complete recovery from neural inactivation (Fig. 1*L*,*M*, blue dots; 101.0 ± 1.4%, vs 100.3 ± 2.0%, before vs after, pairwise comparison; *p* = 0.858; *N* = 12 rats), indicating the suppressive effect was temporary. In the experimental group, HFS-***L*** was applied after lidocaine infusion. LTP was successfully induced even when neurons at recording site ***R*** were temporarily silenced during HFS-***L*** application (Fig. 1*L*,*M*, red dots; two-way RM ANOVA, significant interaction *F*_(1,118)_ = 44.4; *p* < 0.01; 100.9 ± 1.6% vs 139.6 ± 5.4%, before vs after lidocaine infusion and HFS, pairwise comparison; *p* < 0.01; *N* = 12 rats). The results from the control group indicated that there was no rebound effect contributing to the observed LTP after neural suppression. The potentiation of the neural response at site ***R***, despite HFS-***L*** only affecting site ***L*** locally, suggests that local LTP around cell bodies of efferent neurons can enhance signal propagation at distant projection sites without strengthening of synaptic connections at the efferent terminals.

### Visualization of the potentiated neuronal responses at the stimulation site after HFS

**Figure 2.**
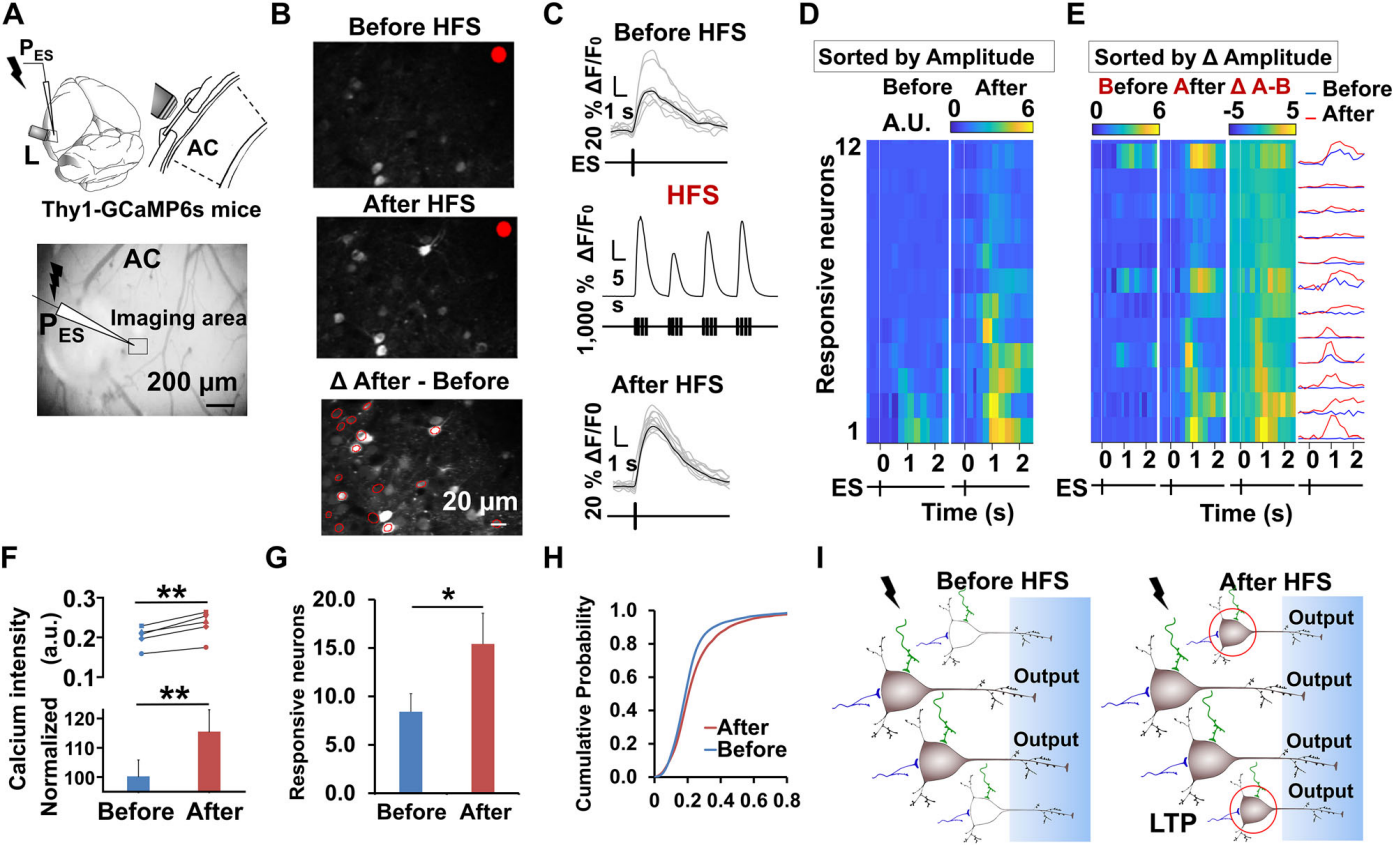
Visualization of the potentiated neuronal responses after HFS. ***A***, Top, Diagram of the imaging experiment. The relative locations of the electrode and the lens in the mouse auditory cortex. Bottom, The locations of the electrode and the imaging area in the auditory cortex (scale bar, 200 µm). ***B***, Calcium imaging of neuronal responses to ES before and after HFS in the recording plane. All neurons that showed significantly increased responses after HFS are marked in red (bottom panel; scale bar, 20 µm). ***C***, The top and bottom panels show the averaged responses of 12 trials before and after the HFS of the red-circled neuron shown in Figure 2*B*. The middle column shows the averaged response of the marked neuron to the HFS. ***D***, Heatmaps show neuronal responses to ES before and after HFS, sorted from strongest to weakest. ***E***, The two heatmaps on the left show the responses to ES before and after HFS, sorted from highest to lowest potentiation levels. The heatmap of column ΔA–B shows changes in neuronal responses to ES after HFS, sorted from largest to smallest. The rightmost panel shows the neuronal calcium signaling responses to ES before and after the HFS. ***F–H***, Bar charts show (***F***) the normalized responses of calcium amplitudes in response to ES (100.0 ± 5.8% vs 115.3 ± 7.7%; **p < 0.01; paired Student's *t* test; *N* = 5 mice; from 157 neurons) and (***G***) the number of neurons that responded to ES after HFS (8.4 ± 1.9 vs 15.4 ± 3.2; *p < 0.05; paired Student's *t* test; *N* = 5 mice). ***H***, The Δ*F*/*F*_0_ cumulative probability before and after HFS. Averaged data for neurons from different mice showed a significantly increased amplitude of calcium imaging after HFS (two-sample Kolmogorov–Smirnov test; *p* < 0.01). ***I***, A schematic illustration shows that more neurons around the stimulation site responded to the probe ES after the HFS. It means the probe ES could produce a stronger output from the stimulation site. Besides the cortical neurons, afferent fibers from other brain regions and cortical fibers could also be activated by the HFS. See Extended Data Figure 2-1 for more details.

The previous results suggested that HFS-induced cortical LTP occurs at the stimulation site rather than the projection terminals at the distant recording site. We inferred that HFS in the neocortex could induce neuroplasticity around the stimulation site, thereby contributing to greater recurrent excitation, causing the same ES to evoke stronger local neural activities post-HFS. To gather more direct evidence, we used two-photon calcium imaging to visualize the HFS-induced changes in the responses of auditory neurons to ES locally. Thy1-GCaMP6s transgenic mice were used as subjects in this experiment and were head-fixed to the stage following induction of a lightly anesthetized state using isoflurane. A stimulation electrode was inserted into the auditory cortex (site ***L***) through a hole in the coverslip, ∼400–600 µm below the dura (Fig. 2*A*). We first examined the proportion of excitatory neurons expressing GCaMP6s indicators through immunohistochemistry and found that 98.8 ± 0.2% of the auditory cortical neurons were colabeled with CamKllα, indicating these neurons were excitatory (Extended Data Fig. 2-1*A*,*B*, a total of 5,785 neurons counted; *n* = 12 slices; *N* = 3 mice). In contrast, almost no inhibitory neurons were labeled (Extended Data Fig. 2-1*C*). Neurons in the recording plane responded to a single ES pulse (Fig. 2*B*, top panel, before the HFS; middle panel, after the HFS; Movie 2-1); the highlighted neurons with red circles (Fig. 2*B*, bottom panel) showed significantly increased responses to the probe ES after HFS. Figure 2*C* displays the calcium signals of a representative neuron (Fig. 2-1*D*, red circled) in response to the probe ES, with the mean amplitude of the calcium signal statistically increased after the HFS (Fig. 2*C*). Across the neuronal population, we observed widespread increases in calcium signal amplitudes in response to the same probe ES after HFS (Fig. 2*D*, with heatmaps sorted by neuronal responses to the ES; Fig. 2*E* left, with heatmaps sorted by changes in neuronal responses after HFS; Fig. 2*E* right, with the heatmap/curves showing the differences in neuronal calcium signals before and after HFS; Fig. 2*F*, 100.0 ± 5.8% vs 115.3 ± 7.7% in calcium intensity, paired Student's *t* test; *p* < 0.01; *N* = 5 mice). Moreover, the number of neurons responding to the ES also increased after HFS (Fig. 2*G*, 8.4 ± 1.9 vs 15.4 ± 3.2, paired Student's *t* test; *p* < 0.05; *N* = 5 mice). Averaged data from different mice showed a significantly increased amplitude of calcium signal after HFS (two-sample Kolmogorov–Smirnov test; *p* < 0.01; Fig. 2*H*). Therefore, HFS induced increases in both the signal amplitudes and the number of neurons responding to the probe ES at the stimulation site. These results provide direct evidence that HFS triggers an enhancement of neuronal responsiveness locally at the stimulation site, leading to stronger output signals evoked by the same ES, which could be regarded as a greater feedforward propagation (Fig. 2*I*).

10.1523/ENEURO.0045-24.2024.f2-1 Figure 2-1 **HFS induced stronger output from the stimulation site.** (**A**) ﻿Immunohistochemistry images illustrating the colocalization of GCaMP6  s and CamKllα (scale bar:20  µm). (**B**) Group data shows the colocalization ratio of CamKllα and GCaMP6  s, N = 3 mice. (**C**) ﻿Immunohistochemistry for GAD67 + and GCaMP6s + cells Thy1-GCaMP6  s mice (scale bar: 20  µm). (**D**) Calcium imaging of neuronal responses to ES from the sampled neuron is shown in Figure 1I (scale bar: 20  µm). (**E**) Confocal images show the colocalization of CCKBR and GCaMP6  s in the auditory cortex of Thy1-GCaMP6  s mice. Download Figure 2-1, TIF file.

### HFS-induced local CCK release enables cortical LTP at the stimulation site

**Figure 3.**
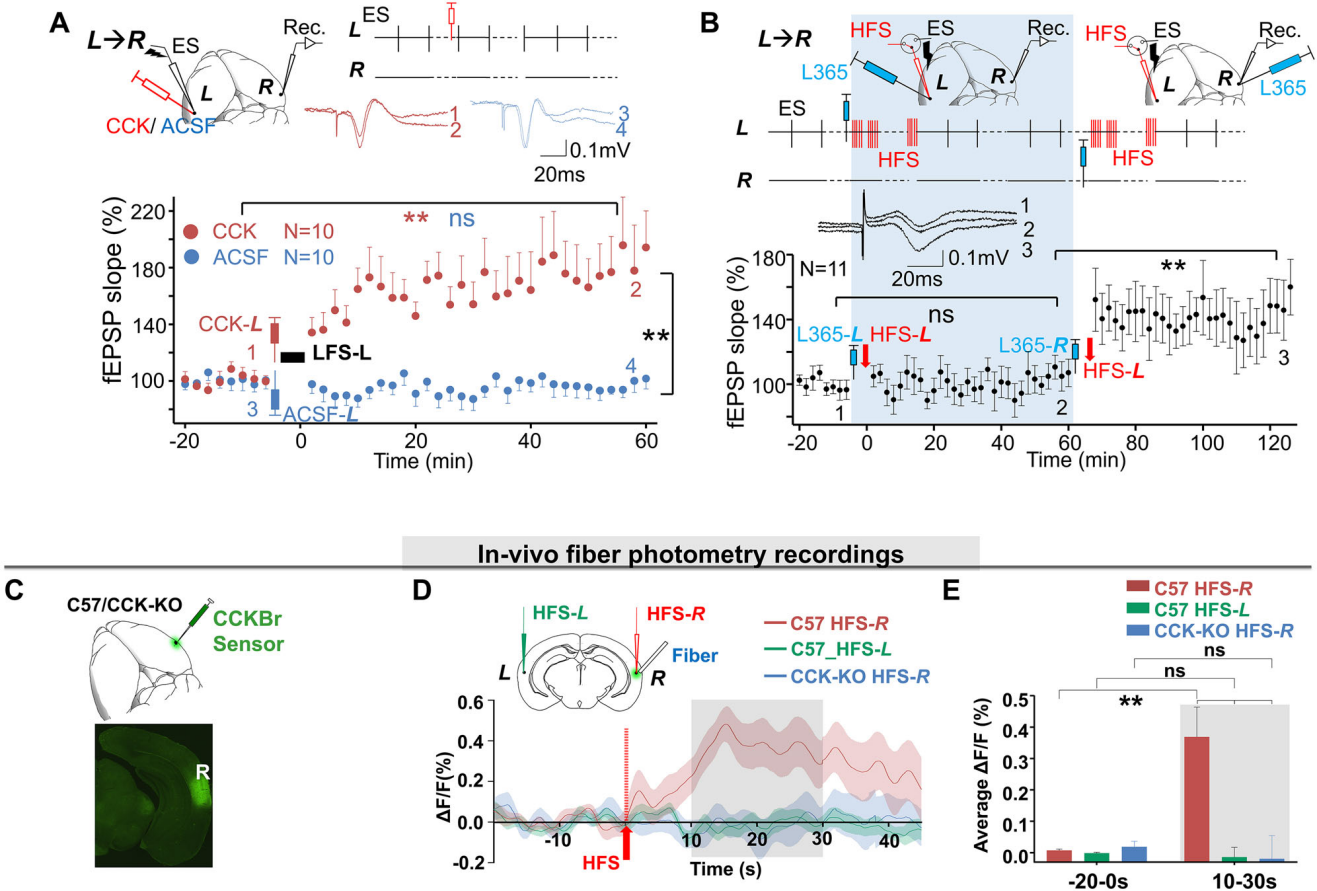
HFS-induced local CCK release enables the cortical LTP at the stimulation site. ***A***, Top, Electrode and drug-injection pipette placement. ES was applied to site ***L*** (ES-***L***). CCK or ACSF was injected at the stimulation site ***L*** (CCK-***L***/ACSF-***L***); representative fEPSP traces of pathway ***L***→***R*** after ACSF injection (1, blue) vs CCK injection (2, red). Bottom, Normalized fEPSP slopes before and after CCK-***L*** (red) or ACSF-***L*** (blue) injections followed by low-frequency ES (LFS-***L***; CCK group, two-way RM ANOVA; **p < 0.01; *N* = 10; ACSF group, ns, two-way RM ANOVA; *p* = 0.685; *N* = 10). ***B***, Top, Electrode and drug-injection pipette placement. A CCKBR antagonist, L365,260, was injected at site ***L*** (L365-***L***) followed by HFS at site ***L*** (HFS-***L***). L365,260 was then injected at site ***R*** (L365-***R***), followed by HFS-***L***, 60 min after the first injection. Probe ES was applied to site ***L*** (ES-***L***) throughout the whole procedure except during injections and HFS. Bottom, Normalized fEPSP slopes before and after each injection followed by HFS-***L*** (before vs after L365-***L*** and HFS-***L***; ns, one-way RM ANOVA; *p* = 1; before vs after L365-***R*** and HFS-***L***; one-way RM ANOVA; **p < 0.01; *N* = 11). ***C***, AAV-syn-CCKsensor was injected into the auditory cortex at site ***R*** of C57 mice or CCK-KO mice. Bottom panel, Virus-infected site ***R***. ***D***, Top, The diagram of the experiment design. HFS was applied to site ***L*** or site ***R*** (HFS-***L*** or HFS-***R***). An optical fiber was placed into site ***R*** to monitor the fluorescence intensity. Bottom, Traces of fluorescence signal of the CCK sensor before and after HFS-***R*** in C57 mice, HFS-***L*** in C57 mice, or HFS-***R*** in CCK-KO mice. Averaged Δ*F*/*F*_0_% was shown as the mean value in the solid line and SEM in the shadow area. The fluorescence increased after HFS-***R*** in C57 mice (red), but the contralateral HFS-***L*** could not induce the increase (green), and neither did HFS-***R*** in CCK-KO mice (blue). ***E***, Bar charts showing the averaged Δ*F*/*F*_0_% from the different groups before and after HFS. (Bonferroni’s multiple-comparison test, before vs after HFS-***R*** in C57, 0.007 ± 0. 004 vs 0.369 ± 0.092; *p* < 0.01; *n* = 18; *N* = 11; before vs after HFS-***L*** in C57, −0.001 ± 0. 003 vs −0.014 ± 0.030; *p* = 1.0; *n* = 18; *N* = 9; before vs after HFS-***R*** in CCK-KO, 0.018 ± 0.018% vs −0.020 ± 0.072; *p* = 1.0; after HFS-***R*** in C57 vs CCK-KO, 0.369 ± 0.092% vs −0.020 ± 0.072; *p* < 0.01; *n* = 19; *N* = 9; after HFS-***R*** vs HFS-***L*** in C57, 0.369 ± 0.092 vs −0.014 ± 0.030; *p* < 0.01).

Based on previous findings, high-frequency optogenetic stimulation of the entorhinoneocortical projections in mice triggers the release of CCK (Li et al., 2023), and the infusion of CCK into the neocortex of rats induced local plastic changes (Li et al., 2014). Therefore, we inferred that local HFS could enable CCK release at the stimulation site, thereby enhancing local neural connectivity and subsequently amplifying the output signal of interhemispheric propagation. If our hypothesis is correct, the application of CCK to site ***L*** should potentiate the neural response to ES-***L*** at the contralateral site ***R***. First, we infused either CCK or ACSF into site ***L*** after baseline testing (Fig. 3*A*). Following the infusion, LFS (0.1 Hz) was applied to site ***L*** to induce both pre- and postsynaptic activation, which is necessary for LTP induction, as described previously. LTP was induced by the CCK infusion but not by the ACSF infusion (two-way RM ANOVA, significant interaction *F*_(1,103)_ = 44.1; *p* < 0.01; 102.1 ± 2.5% vs 183.4 ± 11.8%, before vs after CCK infusion at site ***L***; *p* < 0.01; *N* = 11 rats; 99.7 ± 2.6% vs 96.2 ± 2.7%, before vs after ACSF infusion at site ***L***, pairwise comparison; *p* = 0.705; *N* = 10 rats; Fig. 3*A*). Since only the areas surrounding the drug application site should be affected by CCK, we confirmed that local neuroplasticity triggered by CCK could potentiate the contralateral neural responses through enhanced interhemispheric feedforward propagation.

To confirm that HFS-***L***–induced neuroplasticity is CCK-dependent, we blocked the CCKBR by infusing the antagonist L365,260 into the HFS site ***L*** (L365-***L***) prior to HFS-***L***. This prevented the induction of LTP (99.1 ± 2.2% vs 105.4 ± 3.2%; before vs after L365,260-***L*** infusion and HFS-***L***; pairwise comparison; *p* = 0.353; *N* = 11 rats; Fig. 3*B*). However, when we subsequently infused L365,260 into site ***R*** (L365-***R***, 65 min after L365-***L***), followed by HFS-***L***, the neural responses to ES-***L*** were successfully potentiated (one-way RM ANOVA; significant interaction *F*_(2,108)_ = 33.2; *p* < 0.01; 105.4 ± 3.2% vs 148.0 ± 7.5%; before vs after L365,260-***R*** infusion and HFS-***L***; *p* < 0.01; *N* = 11 rats; Fig. 3*B*). The results indicate that the HFS-***L*** triggers CCK release and enables plasticity specifically at the HFS stimulation site, but not at its interhemispheric projection site. The local CCK release appears to be a key mechanism underlying the HFS-induced cortical LTP observed at the stimulation site.

To directly monitor how cortical HFS triggers CCK release at the stimulation site, we adopted a GPCR activation-based CCK sensor. The release of CCK is indicated by increased fluorescence intensity, which can be measured using fiber photometry. We used C57 mice as subjects and CCK-KO mice as controls. The AAV-syn-CCK sensor was well expressed 4 weeks after virus injection into the auditory cortex (Fig. 3*C*). We applied HFS at the sensor injection site ***R*** (HFS-***R***) in the auditory cortex and monitored the fluorescence intensity at site ***R***. The fluorescence intensity increased significantly following HFS-***R*** in the C57 but not in CCK-KO mice. In contrast, applying HFS at the contralateral site ***L*** (HFS-***L***) did not enhance the fluorescence intensity at site ***R*** (Fig. 3*D*,*E*, averaged Δ*F*/*F*_0_%: two-way ANOVA followed by Bonferroni’s multiple-comparison test; significant interaction *F*_(2,52)_ = 9.90; *p* = 0.0002; before vs after HFS-***R*** in C57, 0.007 ± 0. 004 vs 0.369 ± 0.092; *p* < 0.01; before vs after HFS-***L*** in C57, −0.001 ± 0. 003 vs −0.014 ± 0.030; *p* = 1.0; before vs after HFS-***R*** in CCK-KO, 0.018 ± 0.018 vs −0.020 ± 0.072; *p* = 1.0; after HFS-***R*** in C57 vs CCK-KO, 0.369 ± 0.092 vs −0.020 ± 0.072; *p* < 0.01; after HFS-***R*** vs HFS-***L*** in C57, 0.369 ± 0.092 vs −0.014 ± 0.030; *p* < 0.01). These results demonstrate that HFS in the auditory cortex could trigger CCK release locally at the HFS site, but not contralaterally at the interhemispheric projection site. Given that HFS-induced cortical LTP is CCK dependent as shown in Figure 3*B*, these findings suggest that HFS of the neocortex is more likely to enhance the output signals around the stimulation site than the synaptic connectivity of its efferent terminals at the recording site.

Previous research has shown that cortical HFS in CCK-KO mice cannot induce local LTP, while local administration of CCK rescues this deficit (27). We elucidated that CCK is a crucial factor in HFS-induced local LTP. The other two necessary factors enabling this cortical neuroplasticity are pre- and postsynaptic activation. Thus, this HFS-induced local cortical plasticity can be considered a neo-Hebbian type of plasticity.

### HFS enhances connectivity of long-range afferents to the HFS site

**Figure 4.**
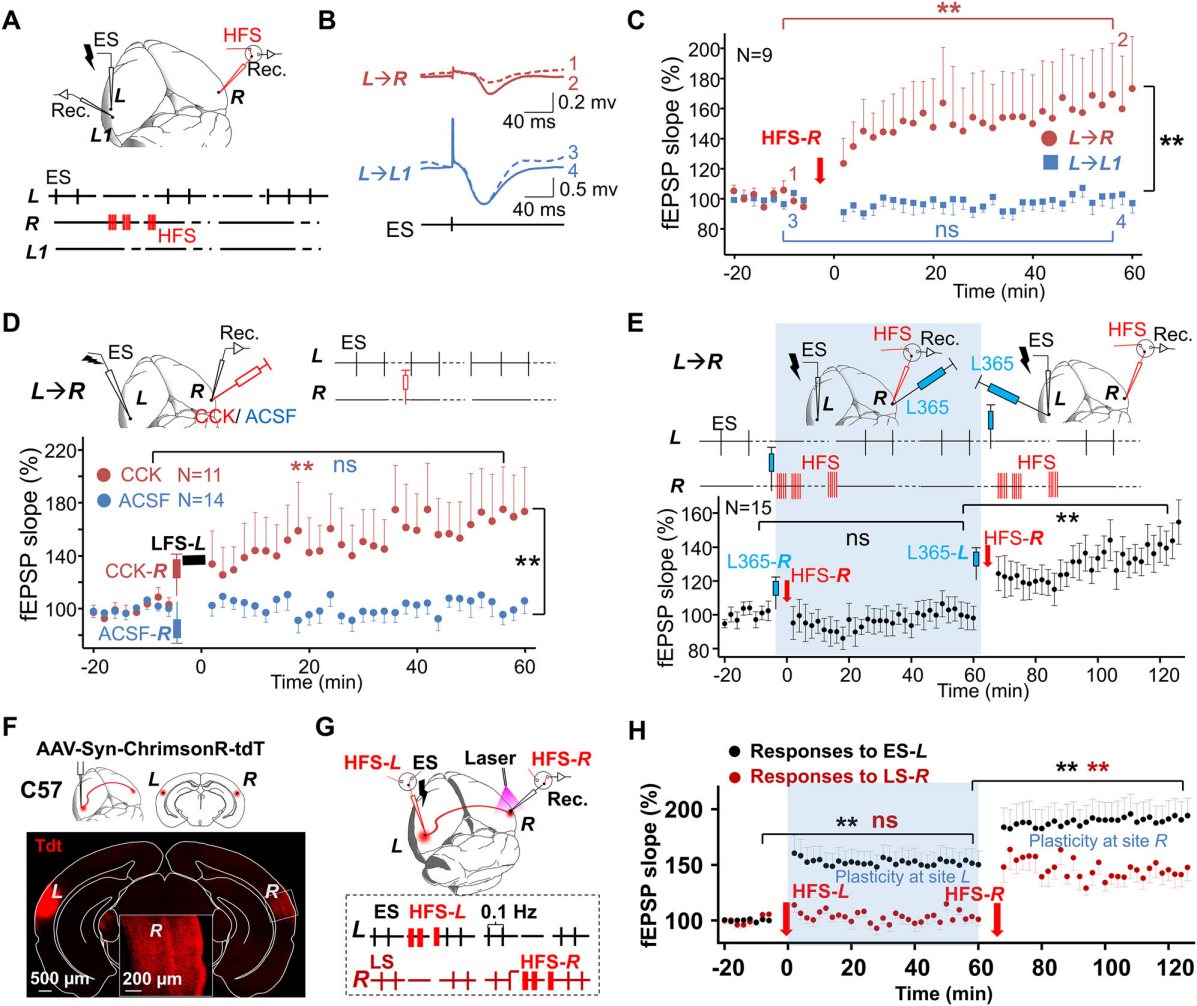
HFS could strengthen the connectivity of long-range afferents to this HFS site. ***A***, Electrode placement. ES was applied to site ***L*** (ES-***L***), and HFS was applied to recording site ***R*** (HFS-***R***). ***B***, Representative fEPSP traces of the connections ***L***→***R*** (top, 1–2) and ***L***→***L1*** (bottom, 3–4) before (dotted) and after (solid) HFS-***R***. ***C***, Normalized fEPSP slopes of the connections ***L***→***R*** (red circle) and ***L***→***L1*** (blue square) before and after HFS-***R*** (***L***→***R***, two-way RM ANOVA; **p < 0.01; ***L***→***L1***, ns, two-way RM ANOVA; *p* = 0.65; *N* = 9). ***D***, Top, Electrode and drug-injection pipette placement. ES was applied to site ***L*** (ES-***L***). CCK or ACSF was injected at the recording site ***R*** (CCK-***R***/ACSF-***R***). Bottom, Normalized fEPSP slopes before and after CCK-***R*** (red) or ACSF-***R*** (blue) injection, followed by LFS-***L*** (CCK group, two-way RM ANOVA; **p < 0.01; *N* = 11; ACSF group, ns, two-way RM ANOVA; *p* = 0.779; *N* = 14). ***E***, Top, Electrode and drug-injection pipette placement. ES was applied to site ***L*** (ES-***L***). L365,260 was injected at site ***R*** (L365-***R*)**, followed by HFS-***R***. L365,260 was then injected at site ***L*** (L365-***L***), followed by HFS-***R***, 60 min after the first injection. Bottom, Normalized fEPSP slopes before and after each injection followed by HFS-***R*** (before vs after L365-***R*** and HFS-***R***, ns, one-way RM ANOVA; *p* = 1; before vs after L365-***L*** and HFS-***R***; one-way RM ANOVA; **p < 0.01; *N* = 15). ***F***, Top, AAV-hSyn-ChrimsonR-tdTomato was injected into the auditory cortex at site ***L*** of C57 mice. Bottom, Images of virus-expressing neurons in injection site ***L*** and site ***R***. Confocal images presenting the afferent fibers were detected in site ***R*** in the enlarged image (scale bars, 500 and 200 µm). ***G***, Top, The diagram of the experiment design. A laser of 620 nm wavelength was used to activate the afferent terminals with opsins expression at site ***R***, which was projected from the contralateral auditory cortex. Glass pipette electrodes at site ***R*** were used to record the field EPSPs evoked by electrical stimulation ES-***L*** and laser stimulations LS-***R***. Bottom, Experimental protocol. ES was applied to site ***L***. Lasers were applied to site R. HFS-***L*** was applied to site ***L***, and then HFS-***R*** was applied to site ***R*** 1 h later. ***H***, Normalized fEPSP slopes in response to ES-***L*** (black) and LS-R (620 nm, red) before HFS-***L***, after HFS-***L***, and after HFS-***R*** (response to ES-***L***, before vs after HFS-***L***, **p < 0.01; two-way RM ANOVA; response to ES-***L***, after HFS-***L*** vs after HFS-***R***; **p < 0.01; two-way RM ANOVA; response to LS-***R***, before vs after HFS-***L***, ns, *p* = 1.0; two-way RM ANOVA; response to LS-***R***, after HFS-***L*** vs after HFS-***R***; **p < 0.01; two-way RM ANOVA; *n* = 16; *N* = 10 mice). See Extended Data Figure 4-1 for more details.

We have demonstrated that cortical HFS induces CCK release at the HFS site and CCK could facilitate neuroplasticity at the CCK application site. Thus, we questioned whether the released CCK could also enhance distant afferent input to the HFS site, besides the local network. We set the recording site ***R*** as the HFS site instead of the ES site ***L***. We examined whether the HFS applied to the site ***R*** (HFS-***R***) could induce the LTP of interhemispheric pathway ***L***→***R***. HFS-***R*** was delivered after performing baseline recording (Fig. 4*A*). The LTP of pathway ***L***→***R*** was readily induced (99.6 ± 2.1% vs 165.7 ± 13.7%; before vs after HFS-***R***; pairwise comparison; *p* < 0.01; *N* = 9 rats; Fig. 4*B*,*C*). Similar to the experiment in Figure 1*H–J*, we also recorded the fEPSP slope of pathway ***L***→***L1***. However, it remained unchanged after HFS-***R*** (99.9 ± 1.5% vs 100.5 ± 2.9%; before vs after HFS-***R***; pairwise comparison; *p* = 0.95; *N* = 9 rats). The difference between the LTP of pathway ***L***→***R*** and pathway ***L***→***L1*** was significant (two-way RM ANOVA, significant interaction *F*_(1,88)_ = 21.0; 165.7 ± 13.7% vs 100.5 ± 2.9%; ***L***→***R*** vs ***L***→***L1***; pairwise comparison; *p* < 0.01). Thus, HFS delivered to the recording site ***R*** successfully induced LTP of pathway ***L***→***R***, not ***L*→*L1***. This would suggest that HFS could strengthen the connectivity of long-range afferents to the HFS site.

10.1523/ENEURO.0045-24.2024.f4-1 Figure 4-1 **HFS could enhance the connectivity of other afferent inputs besides the callosal afferent in a non-specific manner. (A**) Two electrodes were inserted symmetrically into both hemispheres of the auditory cortex, site ***L*** and site ***R***. Auditory stimulus was presented to both ears. HFS was applied to site ***R***. (**B**) Representative fEPSP traces of auditory responses from site ***L*** (upper) or site ***R*** (lower) before (1 and 3) and after (2 and 4) HFS-***R***. (**C**) Normalized fEPSP slopes from site ***L*** (blue spot) or site ***R*** (red block) before and after HFS-***R*** (Site ***L***: ns, two-way RM ANOVA, p = 0.52; Site ***R***: **, two-way RM ANOVA, p < 0.01; N = 12). Download Figure 4-1, TIF file.

Next, we examined whether CCK presented at recording site ***R*** could enable the callosal LTP. Either CCK or ACSF was infused into the recording site ***R*** after baseline testing, and then LFS was applied to site ***L*** (Fig. 4*D*). Indeed, LTP was induced by the CCK infusion at site ***R*** but not by the ACSF infusion (two-way RM ANOVA; significant interaction *F*_(1,123)_ = 24.3; *p* < 0.01; 102.1 ± 2.5% vs 171.7 ± 10.0%; before vs after CCK infusion; pairwise comparison; *p* < 0.01; *N* = 11 rats; 101.3 ± 2.2% vs 103.4 ± 8.9%; before vs after ACSF infusion; pairwise comparison; *p* = 0.820; *N* = 14 rats; Fig. 4*D*). Altogether, these results imply that HFS induces CCK release at the HFS site in the neocortex, enabling neuroplasticity in the surrounding areas, including synaptic strength in the local network and long-range afferents.

Furthermore, we infused the CCKBR antagonist L365,260 into site ***R*** (L365-***R***), followed by HFS-***R*** after baseline testing (Fig. 4*E*). No LTP of pathway ***L***→***R*** was induced (101.7 ± 1.9% vs 100.7 ± 3.2%; before vs after L365-***R*** and HFS-***R***; pairwise comparison; *p* = 1.0; *N* = 15 rats; Fig. 4*E*). We then infused L365,260 into site ***L*** (L365-***L***), followed by HFS-***R***, 65 min after L365-***R*** (Fig. 4*E*). LTP of the pathway ***L***→***R*** was successfully induced (one-way RM ANOVA, significant interaction *F*_(2,148)_ = 48.5; *p* < 0.01; 100.7 ± 3.2% vs 143.1 ± 4.7%; before vs after L365-***L*** and HFS-***R***; *p* < 0.01; *N* = 15 rats; Fig. 4*E*). The findings suggest that HFS enhances the connectivity of callosal afferents to the HFS site through the release of CCK. This result, in combination with the experiment shown in Figure 3*B*, illustrates that HFS whether applied to either the ES site or the recording site potentiates the neural response to ES-***L*** at the recording site ***R***. The application of a CCKBR antagonist at the HFS site blocked LTP, while applying the CCKBR antagonist to the cortex contralateral to the HFS site did not block LTP. Therefore, synaptic plasticity occurs specifically at the HFS site. HFS influences neuroplasticity by inducing the release of CCK locally. These results reconfirm that HFS-induced neocortical LTP is CCK-dependent, encompassing both local LTP and callosal afferent LTP.

We then further questioned whether the cortical HFS could enhance the connectivity of other afferent inputs in a nonspecific manner, besides the callosal afferent. To investigate this, we examined whether the HFS-***R*** potentiated neuronal responses at site ***L*** and site ***R*** to a natural auditory stimulus. An auditory stimulus was presented to both ears of the anesthetized rat, and we recorded the local field potentials of auditory responses from both hemispheres of the auditory cortex in Layer IV (Extended Data Fig. 4-1*A*). After the baseline recording, HFS was applied to site ***R*** but not to site ***L***. As expected, neuronal responses to the auditory stimulus, measured as fEPSPs, were potentiated at site ***R*** but not site ***L*** (two-way RM ANOVA; significant interaction *F*_(1,118)_ = 47.5; *p* < 0.01; site ***L*** recording, 99.4 ± 1.8% vs 101.8 ± 2.5%; before vs after HFS; pairwise comparison; *p* = 0.520; site ***R*** recording, 98.6 ± 1.6% vs 137.4 ± 4.2%; before vs after HFS; pairwise comparison; *p* < 0.01; *N* = 12 rats; Extended Data Fig. 4-1*B*,*C*). This result indicates that the HFS can potentiate various afferent inputs to the HFS site nonspecifically, including those from the ascending auditory pathway, beyond the interhemispheric cortical pathway.

We found that cortical HFS could enhance the local network and the synaptic connectivity of distant afferent input in rats. We then further investigated if this is also the case in mice using an optogenetic method. AAV-hSyn-ChrimsonR- tdTomato virus was injected into the auditory cortex (site ***L***) of C57 mice (Fig. 4*F*). Projections labeled by the virus from site ***L*** were found in the contralateral auditory cortex at site ***R***. Laser stimulation at site ***R*** (LS-***R***) specifically activated the projection terminals of the interhemispheric pathway ***L*** → ***R***, without the influence of the recurrent excitation induced by ES-***L***. Since the ES electrode was inserted into the same location where the virus was injected, LS-***R*** could activate most fiber terminals that ES-***L*** also activated. We applied ES-***L*** and LS-***R*** alternatively and recorded neural responses to ES-***L*** or LS-***R*** at site ***R*** by measuring fEPSPs. HFS-***L*** was first delivered after baseline probe testing (Fig. 4*G*, top panel). The fEPSPs in response to ES-***L*** increased by 51.5 ± 4.0% (pairwise comparison; *p* < 0.01), while fEPSPs to LS-***R*** were not potentiated after HFS-***L*** (increased by 2.4 ± 4.0%; pairwise comparison; *p* = 1.0), indicating that the neuroplasticity did not occur within site ***R***, where synaptic connections of pathway ***L***→***R*** are located, but at the stimulation site ***L***. This is local cortical LTP, not the interhemispheric cortical LTP. We then delivered HFS-***R*** to the recording site ***R*** (∼65 min after HFS-***L***). In contrast, the fEPSPs to both ES-***L*** and LS-***R*** were potentiated after HFS-***R*** this time (response to ES, increased by 40.0 ± 4.5%; pairwise comparison; *p* < 0.01; response to laser, increased by 42.9 ± 6.0%; pairwise comparison; *p* < 0.01.), suggesting that neural plasticity occurred at recording site ***R*** (two-way RM ANOVA; significant interaction *F*_(2,157)_ = 38.1; *p* < 0.01; response to ES, pairwise comparison; response to ES, 100.1 ± 1.2% vs 151.6 ± 3.9% vs 191.6 ± 6.0% before vs after HFS-***L*** vs after HFS-***R***; *n* = 16 from 10 mice; response to laser, pairwise comparison; 100.1 ± 1.2% vs 103.1 ± 3.9% vs 143.7 ± 6.0%; *n* = 16 from 10 mice; Fig. 4*G*,*H*). The true interhemispheric cortical LTP of pathway ***L*** → ***R*** was successfully induced by HFS-***R***.

The results in mice further confirmed that HFS can strengthen the connectivity of interhemispheric afferents to the HFS site (HFS-***R***), but cannot strengthen the connectivity of interhemispheric efferents (HFS-***L***). HFS-induced cortical neuroplasticity does not necessarily occur at synapses at the terminals of its efferent projections but is more likely to occur locally at the HFS site.

The recurrent local LTP on the ipsilateral side of the stimulation site can be interpreted as presynaptic LTP, while the interhemispheric cortical LTP can be viewed as postsynaptic LTP. In Figure 4, applying HFS at recording site ***R*** enhance synaptic connectivity from ***L*** → ***R***. The HFS-***R*** activates projections from ***L*** (as well as entorhinocortical projections, triggering CCK release), leading to LTP in the ***L*** to ***R*** interhemispheric pathway (postsynaptic interhemispheric LTP). Furthermore, the HFS at site ***R*** not only activates afferents from site ***L*** or other regions but also activates local excitatory circuits, enhancing recurrent excitation at site ***R***. This, in turn, would also strengthen signal propagation from ***R*** to ***L*** (as shown in Figs. 1, 3, but with the stimulation and recording locations exchanged). This strengthening of signal propagation from ***R*** to ***L*** is not due to an actual increase in synaptic connectivity from ***R*** to ***L*** (postsynaptic interhemispheric LTP) but rather a result of enhanced local recurrent excitatory circuits at site ***R*** (presynaptic recurrent LTP).

### HFS-induced neuroplasticity modulates behavioral response in a two-alternative choice task

**Figure 5.**
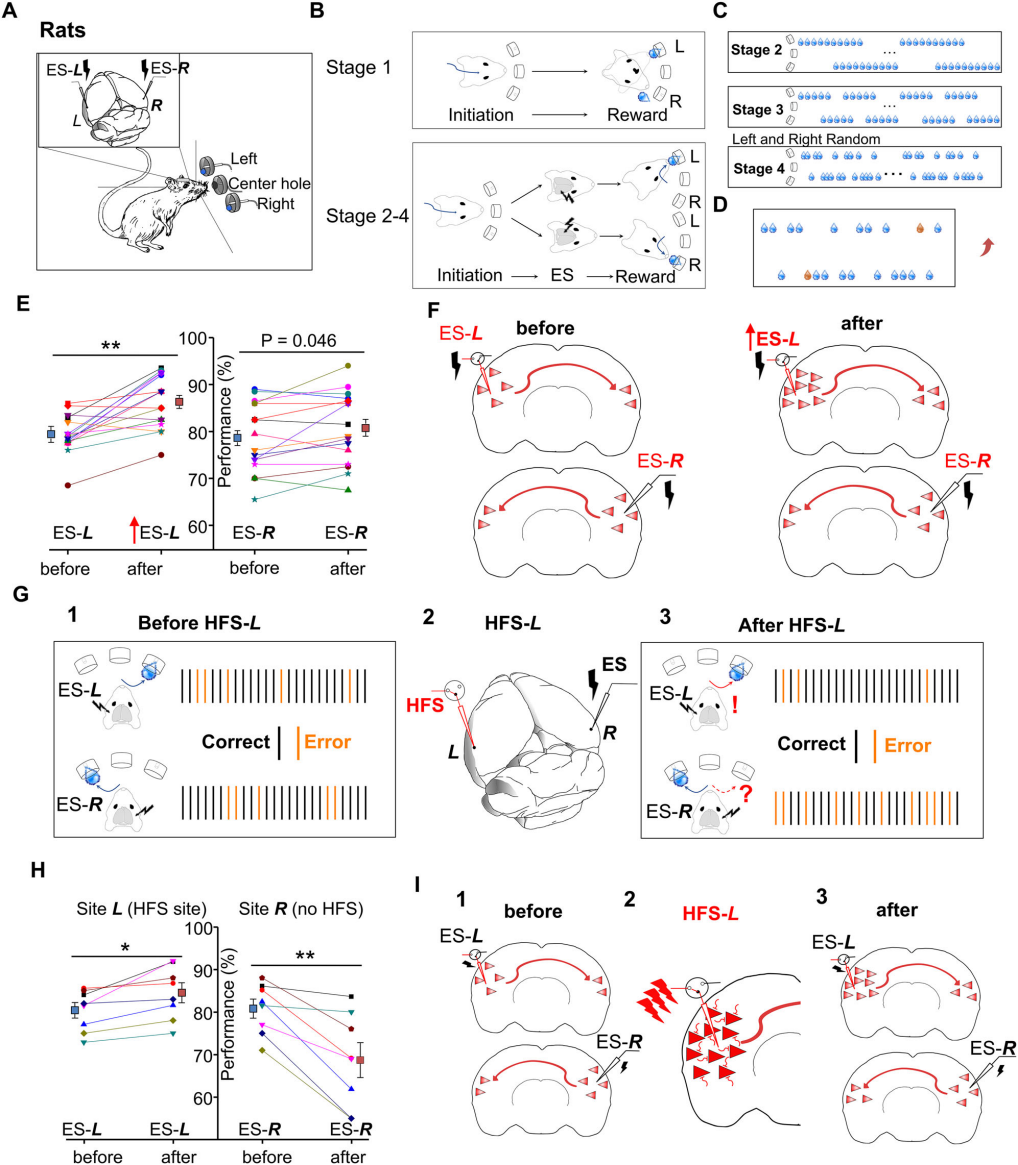
Performance of the two-alternative choice task of the rat changed after HFS-*L*. ***A***, The behavioral apparatus, which includes three holes (right, center, and left). Stimulation electrodes were implanted in both sides of the auditory cortex of rats. A TDT system controlled the delivery of the electrical pulses and the delivery of water rewards. ***B***, The rat initiated each trial by poking its nose into the center hole. During Stage 1, rats could approach either the leftmost hole or the rightmost hole to obtain a water reward after poking its nose into the center hole. During Stages 2–4, after poking its nose into the center hole, the rat would receive one electrical pulse that would be delivered to either hemisphere of the auditory cortex as a cue to indicate the side for water reward delivery. Water was only provided when the rat approached the correct hole for reward retrieval. ***C***, The diagram of the training protocols for Stages 2–4 and the timing of nose-poke detection in the center hole, cue presentation, and reward delivery. During Stage 2, rats learned to use ES-***L*** or ES-***R*** as a cue for reward availability in the right or the left, respectively. Two hemispheres were alternately stimulated between sessions, with 10 trials per session. During Stage 3, hemispheres were alternately stimulated between sessions, with five trials per session. During Stage 4, hemispheres were stimulated in a pseudorandom manner during individual trials. ***D***, An example of rat performance after achieving a success rate of >85%. Water drops in blue indicate that the rat succeeded in reward retrieval, while orange indicates a failed trial. ***E***, Performance before and after the ES-***L*** current was increased by 20%. Left panel, The correct rate toward the ES-***L*** cue. 79.4 ± 1.1% to 86.3 ± 1.5%; **, paired Student's *t* test. Right panel, The correct rate toward the ES-***R*** cue which the current did not change before versus after. 78.6 ± 1.9% to 80.8 ± 2.0%; *p* = 0.046; paired Student's *t* test. *n* = 16; *N* = 6. ***F***, A model of the increased current of the ES-***L*** elevated the neuronal responses in the left hemisphere, resulting in an improved performance toward the cue of ES-***L***. ES-***R*** remained unchanged, so the improvement toward the cue ES-***R*** was minimal but statistically significant. ***G***, The experimental paradigm. ***G*1**, Before the HFS treatment, the rat's success rate was adjusted to ∼80% for each side by lowering the cue current applied to both hemispheres (see Extended Data Fig. 5-1*B* for the current-dependent success rate function). In response to ES-***L***, the rat poked the rightmost hole to retrieve a water reward, and in response to ES-***R***, the rat poked the leftmost hole (black bars). Orange bars indicate that the rat approached the wrong hole. ***G*2**, HFS applied to the left hemisphere, marked as HFS-***L***. ***G*3**, After HFS-***L*** treatment, the rat showed an increased success rate when ES-***L*** was presented and a reduced success rate when ES-***R*** was presented. Black bars, successful trials; orange bars, failed trials. ***H***, Averaged success rates of all rats (*N* = 8) before (red) and after (blue) HFS-***L*** in response to the ES cue. Left, Success rates when the ES-***L*** cue was applied; 80.4 ± 1.8% vs 84.5 ± 2.3%; paired Student's *t* test; *p = 0.011. Right, Success rates when the ES-***R*** cue was applied; 80.8 ± 2.2% vs 68.7 ± 4.1%; paired Student's *t* test; **p < 0.01. ***I***, A model shows the ES cue-triggered neural activity before and after HFS-***L***. ***I*1**, Some neurons in the left hemisphere and a smaller amount of neurons in the right hemisphere respond to ES-***L*** (top). Some neurons in the right hemisphere and a smaller amount of neurons in the left hemisphere respond to ES-***R*** (bottom). ***I*2**, HFS-***L*** with higher current activated terminals and neurons at the stimulation site and induced plasticity at site ***L***. ***I*3**, After HFS-***L***, the ES-***L*** evoked increased activity in the left auditory cortex, leading to an increased success rate (top), while ES-***R*** evoked the same level of activity in the right hemisphere and increased activity in the left hemisphere, leading to difficulty determining the cue for reward retrieval and a reduced success rate (bottom). See Extended Data Figure 5-1 for more details.

In the final experiment, we designed a behavioral task to examine the HFS-induced neuroplasticity using a two-alternative choice paradigm. This behavioral paradigm was similar to that previously reported (Zhang et al., 2020). Two stimulation electrodes were bilaterally implanted into the auditory cortices of the rat at the previously described sites ***L*** and ***R***. Each trial was initiated by the rat poking its nose into the center hole of the three-hole apparatus (Fig. 5*A*). An ES cue was delivered to one hemisphere of the auditory cortex, indicating water availability on the opposite side. A water reward was provided only when the sensors at the indicated hole detected the rat's arrival within 2 s of the ES cue (Fig. 5*B*).

10.1523/ENEURO.0045-24.2024.f5-1 Figure 5-1 **HFS-induced local neuroplasticity modulates behavioral response in a two-alternative choice task.** (**A**) The success rate of an example rat during training from Stages 2 to 4. (**B**) Representative relationship between input currents and performance after the rat was successfully trained. We adjusted the stimulation current, choosing a current that resulted in a success rate of approximately 75–85% as the stimulation current for the next step of the experiment. (**C**) HFS treatment on an anesthetized rat. The recording electrode was placed in the left hemisphere of the auditory cortex, while the stimulation electrode was placed in the right hemisphere. HFS was applied to the site ***L*** (HFS-***L***). **(D**) Single fEPSP traces representing the ***R***→***L*** connection (blue) and ***L***→***R*** connection (red). (**E**) Normalized fEPSP slopes before and after each HFS. Probe ES was applied to the right site (ES-***R***), while HFS was applied to the left side (HFS-***L***). HFS-***L*** was delivered 3 times in a 30-minute interval. The experimental protocol is shown above the time course of the fEPSP slope. (**F**) fEPSP traces represent the pathway ***R***→***L*** before and after each HFS-***L***. (**G**) Bar charts showing the slope of fEPSPs before and after each HFS-***L*** (before (1) vs. after (2) 1^st^ HFS-***L***, 104.9 ± 3.2% vs. 141.8 ± 4.1%, **, one-way RM ANOVA, p < 0.01; before (2) vs. after (3) 2^nd^ HFS-***L***, 141.8 ± 4.1% vs. 171.4%±5.9%, **, one-way RM ANOVA, p < 0.01; before (3) vs. (4) after 3^rd^ HFS-***L***, 171.4 ± 5.9% vs. 181.5 ± 11.5%, ns, one-way RM ANOVA, p = 0.144); n = 7. (**H**) The HFS protocol. Download Figure 5-1, TIF file.

**Movie 2-1.** Calcium imaging of neuronal responses to ES, in the recording plane before and after HFS. [View online]

**Movie 5-1.** An example of rat behavior performance before HFS-***R***. A nose-poking to the center hole (lasting for 300–600 ms) triggered the delivery of ES-***L*** or ES-***R***, indicating water availability on the opposite side. The computer paused the task for 3 s, when it detected a wrong choice in response to the ES cue. [View online]

**Movie 5-2.** An example of rat behavior performance after HFS-***R***. The task performance in response to the ES-***L*** cue was severely affected following HFS-***R***. In contrast, task performance improved in response to the ES-***R*** cue. [View online]

The training process was divided into four stages. In Stage 1, the rat received a water reward at either the left or right hole after initiating the trial at the center hole. In Stage 2, ES cues were alternately applied to the left and right hemispheres in blocks of 10 trials each (Fig. 5*B*). In Stage 3, each block consisted of five trials instead of 10. In Stage 4, the ES cue was applied to either the left or right hemisphere in a pseudorandom manner (Fig. 5*C*).

After training, the rat successfully associated the cues with the water rewards, achieving a success rate of over 90%, as shown in Figure 5*D* (Extended Data Fig. 5-1*A* shows an example of training progress). Upon completion of training, the success rate could be manipulated by adjusting the stimulation current (Extended Data Fig. 5-1*B*). For subsequent experiments, we reduced the stimulation current to achieve a success rate of ∼80% (Extended Data Fig. 5-1*B*).

During the two-alternative choice task, the ES cue applied to one side initially activated neurons near the stimulation site and then induced secondary neuronal responses on the contralateral side due to interhemispheric projections. The rat distinguished the direct ES-evoked neural responses in one hemisphere from the secondary response, as evidenced by its correct retrieval of the water reward.

We first examined how increasing the stimulation current of the ES cue in one hemisphere affected the success rate. We increased the current of the ES cue applied to the left hemisphere (ES-***L***) by ∼20% while keeping the current of the ES cue applied to the right hemisphere (ES-***R***) constant. As shown in Figure 5*E*, the success rate improved from 79.4 ± 1.1% to 86.3 ± 1.5% in response to the increased ES-***L*** current (*p* < 0.01; paired Student's *t* test; *n* = 16 from six rats). Meanwhile, the task performance in response to the unchanged ES cue applied to the right hemisphere (ES-***R***) showed minimal improvement, from 78.6 ± 1.9% to 80.8 ± 2.0; *p* = 0.046; paired Student's *t* test; *n* = 16 from six rats; Fig. 5*E*).

The increased current of the ES-***L*** potentiated the neuronal responses at site *L*, leading to improved performance toward the ES-***L*** cue, as illustrated in the model in Figure 5*F*. The improvement toward the ES-***R*** cue was minimal but statistically significant.

Next, we investigated whether the HFS at one hemisphere enhances local neural connectivity at the stimulation side by evaluating animals' behavioral performance in the two-alternative choice task. After baseline performance testing (Fig. 5*G*1), we anesthetized the rats with pentobarbital and applied HFS at site ***L*** (HFS-***L***) three times (Fig. 5*G*2; Extended Data Fig. 5-1*C*–*G*; HFS protocol: Extended Data Fig. 5-1*H*).

#### Performance in response to ES-***L***

According to our previous result in Figure 2, HFS-***L*** could enable local neuroplasticity and increase the number of neurons responding to the ES-***L*** at site ***L*** (Fig. 5*I*1,3, top). Consequently, the rats should demonstrate improved performance on the two-alternative choice task in response to ES-***L*** after HFS-***L***. After recovering from anesthesia the next day, the rat's success rate in response to ES-***L*** indeed increased from 80 to 88% in the example shown in Figure 5*G*1 and 3 (top panel). Across all trained animals, the success rate in response to ES-***L*** on the two-alternative choice task improved slightly after HFS-***L***. (To make our explanation easier, we set the left side as the hemisphere that received HFS; Fig. 5*H*, left; 80.4 ± 1.8% vs 84.5 ± 2.3%; before vs after treatment; *p* = 0.011; paired Student's *t* test; *N* = 8).

#### Performance in response to ES-***R***

While the rats were anesthetized, the HFS-***L*** was applied three times. The neural response in the left hemisphere, in response to ES-***R***, was potentiated from 104.9 ± 3.1% to 141.8 ± 4.1%, 171.4 ± 5.9%, and 181.5 ± 10.5% of the baseline after each application of HFS-***L*** (Extended Data Fig. 5-1*C*–*G*). The interhemispheric synaptic connectivity of ***R***→***L*** at site ***L*** was enhanced after the application of the HFS-***L*** (Fig. 5*I*2, similar to the experiment in Fig. 4). This enhancement should be detectable during the response to the ES-***R***, with more neurons on the left side demonstrating increased activity in response to the ES-***R***, while the neuronal responses in the right hemisphere should remain unchanged (Fig. 5*I*3,1). We therefore deduced that the performance of the rat on the two-alternative choice task would decline in response to the ES-***R***, as the increased activity in the left hemisphere could confuse the rat in selecting the correct choice. As expected, the success rate in response to ES-***R*** decreased from ∼80 to 60% following the application of HFS-***L*** in the example (Fig. 5*G*1,3, bottom). In the group data, the success rate in response to ES-***R*** decreased significantly after HFS-***L*** (paired Student's *t* test; before vs after treatment; 80.8 ± 2.2% vs 68.7 ± 4.1%; *p* < 0.01; *N* = 8; Fig. 5*H*, right).

This experiment confirmed that HFS-induced neuronal plasticity affected behavioral performance by reinforcing the neural connectivity at the stimulation site. An example of the performance before and after HFS-***R*** is shown in Movies 5-1 and 5-2.

In summary, the task performance in response to the ES cue, which was applied to the hemisphere contralateral to the HFS site, was severely affected following HFS. The neurons in site ***L*** demonstrated an increased response to the ES-***L*** after HFS-***L***, similar to the improved task performance observed with increased current stimulation in Figure 5*E* (Fig. 5*I*1,3). In contrast, the strengthened interhemispheric synaptic connectivity from the contralateral hemisphere (site ***R***) to the HFS site ***L*** was the likely reason for the worsened performance in response to the cue ES-***R***. Although the cue ES-***R*** resulted in the same level of activation at site ***R*** both before and after HFS-***L***, the secondary activation of the interhemispheric projection at site ***L*** was elevated, confusing the rat and making it difficult to distinguish the activation of neurons on one side from the other.

## Discussion

In this study, we discussed two easily overlooked mechanisms of HFS-induced cortical plasticity.

Firstly, upon application of HFS to the cortical region, nearby neurons are activated, some of which extend projections to distant areas. The conventional wisdom suggests that HFS-induced LTP occurs at the synapses on the projection terminals (distant recording sites) of these activated neurons. However, our research challenges this notion, as the high-frequency activation of many cortical neurons does not necessarily trigger the release of CCK at their synaptic terminals (Fig. 3*C*–*E*; Li et al., 2023; Sun et al., 2024), which is a pivotal factor for the formation of synaptic plasticity. Therefore, synaptic strengthening does not occur at the projection terminals (recording sites) of these neurons. Instead, HFS in the cortical region triggers local CCK release at the site of stimulation, thereby strengthening the connectivity of nearby excitatory circuits and intensifying recurrent excitation. Consequently, this amplifies the output signal for the same ES at the stimulation site, subsequently enhancing the neural responses recorded at the distant recording site. Our findings demonstrate that cortical HFS-induced local plasticity augments the signal propagation of callosal efferents, which is mediated by CCK release at the HFS site that boosts the local excitatory network; the subsequent enhanced recurrent network then amplifies the feedforward output signal of the callosal pathway.

Secondly, while cortical HFS may not strengthen the synapses at the distant efferent terminals of the activated neurons, it can activate the afferent fibers originating from distant regions. According to the classical Hebbian rule, pre- and postsynaptic coactivation fosters neuroplasticity, suggesting that cortical HFS could enable neuroplasticity at the stimulation site, specifically at synapses between activated afferent fiber terminals and activated postsynaptic neurons. However, this alone is insufficient. Our results indicate that CCK, which is incapable of homosynaptic release from callosal (cortical) projections, is indispensable for this process, supplementary to the traditional Hebbian rule. When complemented by the locally released CCK due to HFS, this interplay prompts synaptic plasticity at the terminals of these afferents.

In essence, synaptic plasticity induced by cortical HFS primarily occurs near the HFS site, incorporating the reinforcement of excitatory recurrent circuits among adjacent neurons and the plasticity of synapses formed by distant afferent fibers. This is attributed to HFS's ability to induce local CCK release and activate both the presynaptic and postsynaptic responses nearby, embodying a neo-Hebbian form of synaptic plasticity mechanism.

Although HFS triggered CCK release locally (Fig. 3*D*,*E*), we did not confirm which afferents account for this release, as HFS could potentially activate multiple afferents. Previous studies suggest that CCK-positive neurons in the entorhinal cortex project extensively into the neocortex (Li et al., 2014; Chen et al., 2019; Sun et al., 2024). Electrical stimulation of the entorhinal cortex or optogenetic stimulation of the entorhinocortical projections facilitated CCK-dependent neuroplasticity occurring in the neocortex (Li et al., 2014, 2023; Chen et al., 2019; Zhang et al., 2020; Sun et al., 2024). The high-frequency optogenetic activation of the entorhinoneocortical projections triggered CCK release (Li et al., 2023). Hence, we believe the entorhinal CCK afferent contributes significantly to the cortical CCK release. Therefore, the cortical HFS-induced plasticity is likely to be modulated by entorhinal projections through CCK release.

Our fiber photometry data showed that HFS of the contralateral callosal projection neurons could not induce CCK release through the auditory callosal pathway (Fig. 3*D*,*E*). This explains why high-frequency activation of the callosal projection, whether through contralateral HFS (Fig. *4F*–*H*, HFS-***L***) or high-frequency optogenetic stimulation (Li et al., 2023), was unable to strengthen the interhemispheric connectivity. This phenomenon is not unique to corticocortical plasticity induction. High-frequency optogenetic stimulation of entorhinoneocortical projection, rather than the visuoauditory cortical projection, was required to induce the plasticity of visuoauditory inputs (Sun et al., 2024). RNAscope analysis confirmed that the expression level of CCK was significantly higher among cortical-projecting neurons in the entorhinal cortex than in the visual cortex (Sun et al., 2024). Previous reports indicate that both interhemispheric and visuoauditory cortical LTP can only be induced by heterosynaptic activation of entorhinal projection, and not by the homosynaptic activation alone (Li et al., 2023; Sun et al., 2024). This heterosynaptic modulation of plasticity may be a general principle applicable to other corticocortical pathways as well (Bailey et al., 2000; Chistiakova and Volgushev, 2009; Chistiakova et al., 2014). The neocortex is essential for long-term memory storage (Alvarez and Squire, 1994; McClelland et al., 1995; Squire and Alvarez, 1995; Bontempi et al., 1999; Chklovskii et al., 2004; Maviel et al., 2004; Frankland and Bontempi, 2005; Yamashita et al., 2009; Bero et al., 2014; Dalmay et al., 2019). However, traditional Hebbian-type homosynaptic plasticity tends to produce runaway dynamics of synaptic weights, amplifying correlations in neural circuits and creating positive feedback or negative feedback loops (Chen et al., 2013). The neo-Hebbian rule mitigates these instability issues in memory formation and retrieval by constraining the plasticity requirements: the modulation of neuromodulators, such as dopamine or CCK, supplementaryto pre- and postsynaptic activity (Lisman et al., 2011; Frémaux and Gerstner, 2015; Gerstner et al., 2018). Without the heterosynaptic CCK release modulation from entorhinal projection, the plasticity of cortical callosal input or visuoauditory cortical input cannot be established (Li et al., 2023; Sun et al., 2024). This conclusion aligns with the understanding that the hippocampus system influences memory encoding in the neocortex (Alvarez and Squire, 1994; McClelland et al., 1995; Squire and Alvarez, 1995; Bontempi et al., 1999; Maviel et al., 2004; Frankland and Bontempi, 2005; Yamashita et al., 2009; Bero et al., 2014) and the entorhinal cortex, acting as the gateway from the hippocampus to the neocortex, could facilitate cortical neuroplasticity via CCK release (Buzsáki, 1996; Remondes and Schuman, 2004; Li et al., 2014; Chen et al., 2019; Ohara et al., 2023).

CCK, the most abundant neuropeptide in the brain, is a key chemical for neural plasticity and is associated with learning and memory (REHFELD, 1978; Moran and Schwartz, 1994; Nomoto et al., 1999; Hadjiivanova et al., 2003; Lo et al., 2008; Li et al., 2014; Chen et al., 2019). CCK knock-out mice or CCK AB receptor knock-out mice exhibit deficits in cortical LTP induction and associative learning (Chen et al., 2019; Li et al., 2023). In this study, we segregated the stimulation and recording sites. The infusion of CCK at either the stimulation site ***L*** or the recording site ***R*** potentiated the signal propagation of the callosal pathway (Figs. 3*A*, 4*D*), confirming that in the presence of CCK, LFS can induce LTP at the CCK application site through pre- and post-coactivation. The injection of a CCKBR antagonist only at the HFS site (Figs. 3*B*, 4*E*, either ***L*** or ***R***) blocked the LTP induction. These results indicate that CCK is a crucial for HFS-induced cortical LTP, serving as a third requirement in neo-Hebbian–type plasticity. CCK-A receptor and CCKBR are coupled to the G-protein type q, which triggers intracellular calcium signaling through the activation of phospholipase C (Lee et al., 1993; Yule et al., 1993, 1999; Detjen et al., 1997; Dufresne et al., 2006). In the central nervous system, CCK also has been reported to enable extracellular calcium influx through L-type or N-type voltage–sensitive calcium channels in neurons (Miyoshi et al., 1991; Zhang et al., 2002). The calcium elevation of postsynaptic neurons can subsequently facilitate AMPA receptor trafficking and postsynaptic plasticity (Lynch et al., 1983; Malenka et al., 1988; Auerbach and Segal, 1994; Wang et al., 1996; Berridge, 1998; Yang et al., 1999; Zucker, 1999; Bredt and Nicoll, 2003). The calcium-based plasticity model explains spike-timing–dependent synaptic plasticity (Karmarkar and Buonomano, 2002; Gerkin et al., 2010; Graupner and Brunel, 2012; Inglebert and Debanne, 2021). Calcium influx through NMDA receptors is thought to be critical in synaptic plasticity (Bliss and Collingridge, 1993; Malenka and Bear, 2004; Lau et al., 2009). Interestingly, while the application of an NMDA receptor antagonist could block HFS-induced cortical LTP, the LTP was still induced after NMDA receptor blockade in the presence of CCK (Chen et al., 2019). This emphasizes the crucial role of CCK in calcium signaling and plasticity. Given the importance of calcium in synaptic plasticity induction, variations in calcium concentration in vitro or in vivo may influence LTP induction results (Inglebert et al., 2020; Chindemi et al., 2022). The classical Hebbian LTP induction in the rat hippocampus was greatly affected by calcium (Inglebert et al., 2020). The nonphysiological higher calcium concentrations in some in vitro slice studies may explain why interhemispheric cortical LTP could be induced in a classical Hebbian manner rather than a neo-Hebbian manner with CCK modulation (Sah and Nicoll, 1991; Petrus et al., 2019). In addition to CCK, neurotransmitters such as dopamine and norepinephrine participate in the modulation of LTP and synaptic plasticity to varying degrees (Katsuki et al., 1997; Tully et al., 2007; Lisman et al., 2011). These mechanisms are crucial for the formation and consolidation of learning and memory (Hu et al., 2007; Kempadoo et al., 2016; Takeuchi et al., 2016). The interplay of these neuromodulators and their regulation of synaptic plasticity warrant further in-depth investigation.

The neocortex is a complex cellular system with six horizontal layers, each exhibiting distinct anatomical and functional properties through strongly interconnected cortical neurons both within and across layers (Douglas and Martin, 2004). In the present electrophysiology studies, the stimulation and recording electrodes were placed in Layer V of the left and right auditory cortex. The interhemispheric signal propagated from the contralateral mirrored location was recorded in the form of fEPSP (Fig. 1*A*–*E*). Since callosal projections also originate from Layers II/III in addition to Layer V (Kawaguchi, 1992; Petreanu et al., 2007) and given the extensive mutual excitatory projections exist between Layers II/III and Layer V (Binzegger et al., 2004; Shepherd and Svoboda, 2005; Yoshimura et al., 2005; Lefort et al., 2009; Hooks et al., 2013), the signal propagation from contralateral Layers II/III may also contribute to our result. We did not specifically differentiate between layers II/III and V regarding their contributions to local excitatory neural circuits and recurrent excitation. In our two-photon imaging experiments, both the depth of stimulation (layers IV) and recording (layers II/III) were at shallower depths compared with those utilized in the electrophysiological experiments. This methodological distinction may have impacted the interpretation of our results and should be considered in future investigations.

While our analysis focused on the slope of fEPSP, the potential contribution of indirect projection should not be overlooked. In this study, the HFS-***L*** enhanced the local excitatory network, and the resulting increased recurrent excitation amplified interhemispheric signal propagation. Notably, the fEPSP recorded at site ***R*** in response to contralateral ES-***L*** comprises a blend of monosynaptic excitation, polysynaptic excitation, and even polysynaptic inhibition, given that the callosal projection also targets inhibitory interneurons (Bloom and Hynd, 2005; Rock and Junior Apicella, 2015). The balance between excitation and inhibition is critical for the functional organization of the neocortex. Due to the limitations of our methodology, we only focused on how CCK influences excitatory neurons. The effects of CCK on cortical GABAergic interneurons remain elusive. We acknowledge that our explanation is not comprehensive and further research is needed to fully elucidate the complex mechanisms governing cortical plasticity.

Another limitation of our work is the use of different anesthetics based on the specific requirements of each experimental procedure. Urethane was primarily chosen for in vivo extracellular recordings due to its ability to maintain stable physiological conditions over extended periods without the need for supplementary doses, supporting consistent monitoring of neuronal responses and plasticity. In contrast, isoflurane was used in two-photon calcium imaging experiments to focus on immediate changes in neuronal responses and allowing us to examine the effects of cortical HFS in a lightly anesthetized state. For surgical procedures and virus injections, pentobarbital was selected for its shorter duration, facilitating animals' quick recovery and enabling assessment of HFS-induced plasticity effects in a wakeful state during behavioral studies. These variations in anesthetic use highlight the importance of considering how different anesthetics might influence neural activity and plasticity. Urethane is known for its association with cortical up–down states (Steriade et al., 1993a,b), which may affect the effectiveness of ES. Cortical up states are intrinsically driven by recurrent excitation (Steriade et al., 1993b; Sanchez-Vives and McCormick, 2000; Timofeev et al., 2000), with network dynamics stabilized by balanced increases in inhibition (Sanchez-Vives and McCormick, 2000; Shu et al., 2003; Haider et al., 2006; Bartram et al., 2017). HFS augmented the local recurrent excitation networks, thereby potentially enhancing the balance of excitation to tend to self-sustained activity (up states), offering another explanation. Future research should explore these dynamics further to improve our understanding of how anesthetic choice impacts cortical plasticity.

In summary, we found that CCK is the crucial modulator for cortical plasticity. Importantly, CCK can only be released at the HFS site from other efferents, but not from cortical neurons in a homosynaptic manner (Fig. 3*C–E*, cortical HFS enabled CCK release locally, whereas the HFS of contralateral callosal projection neurons did not enable CCK release). Thus, cortical HFS exerts influence on long-range cortical efferent via changes at the HFS location rather than at their projection terminals. Meanwhile, the HFS-released local CCK also strengthened the long-range afferent synapses to the HFS site. Collectively, these findings reveal that a CCK-dependent neo-Hebbian mechanism underlines cortical plasticity.

## Synthesis

Reviewing Editor: Nathalie Ginovart, University of Geneva Decisions are customarily a result of the Reviewing Editor and the peer reviewers coming together and discussing their recommendations until a consensus is reached. When revisions are invited, a fact-based synthesis statement explaining their decision and outlining what is needed to prepare a revision will be listed below. The following reviewer(s) agreed to reveal their identity: NONE.

The authors have engaged in a constructive revision of their manuscript, and this revised version is significantly improved compared to the original submission. However, before final acceptance, authors must comply with eNeuro's policy regarding studies using custom code. The code must be available upon acceptance and publication of the manuscript. As per eNeuro's Instructions to authors, code files should be deposited in a suitable repository such as GitHub, ModelDB, BioModels, CellML, or Visiome. The "Code Accessibility" section should therefore indicate how the code can be accessed and provide a repository link, including any accession numbers or restrictions, as well as the type of computer and operating system used to run the code.

Please find below reviewer 1's comment:

Reviewer 1:

The authors have responded to my comments on the initial submission well. No further comments.
