# Supplementary figures and images for: Cortical HFS-Induced Neo-Hebbian Local Plasticity Enhances Efferent Output Signal and Strengthens Afferent Input Connectivity
Source: eNeuro. 2025 Feb 3;12(2):ENEURO.0045-24.2024. doi: 10.1523/ENEURO.0045-24.2024 (PMC11810566; doi:10.1523/ENEURO.0045-24.2024)

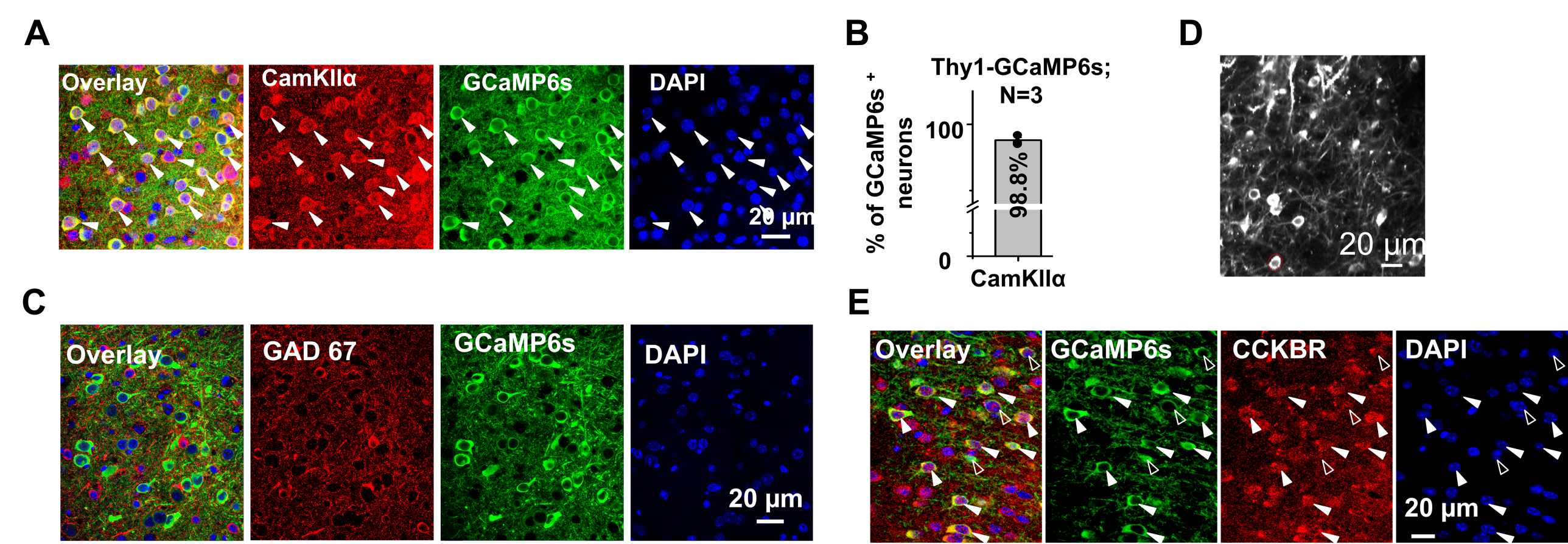

Supplement: Figure 2-1 — HFS induced stronger output from the stimulation site. (A) ﻿Immunohistochemistry images illustrating the colocalization of GCaMP6 s and CamKllα (scale bar:20 µm). (B) Group data shows the colocalization ratio of CamKllα and GCaMP6 s, N = 3 mice. (C) ﻿Immunohistochemistry for GAD67 + and GCaMP6s + cells Thy1-GCaMP6 s mice (scale bar: 20 µm). (D) Calcium imaging of neuronal responses to ES from the sampled neuron is shown in Figure 1I (scale bar: 20 µm). (E) Confocal images show the colocalization of CCKBR and GCaMP6 s in the auditory cortex of Thy1-GCaMP6 s mice. Download Figure 2-1, TIF file. [file eneuro-12-ENEURO.0045-24.2024-s003.tif]

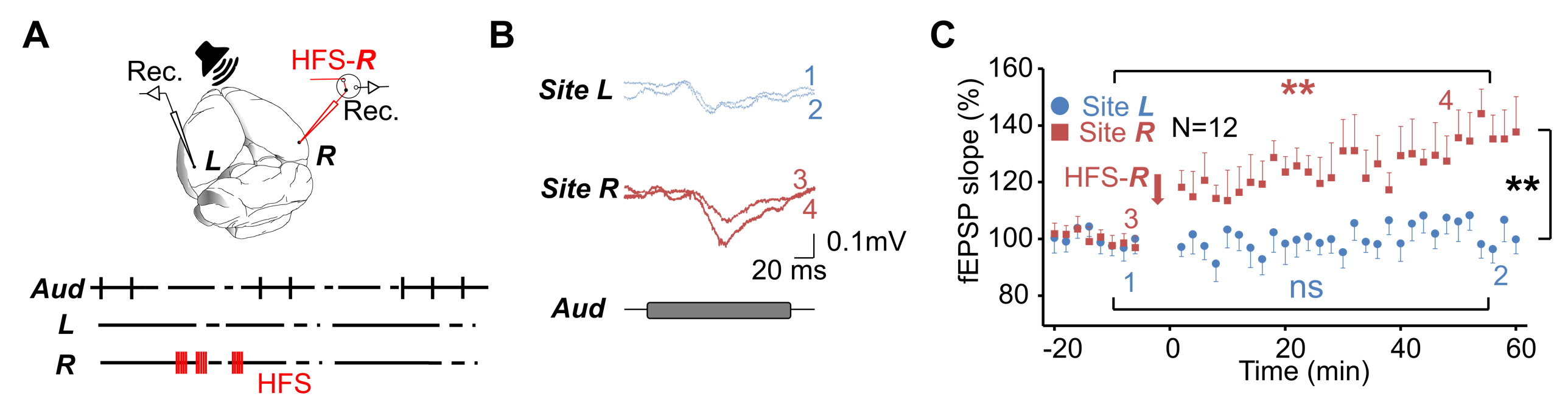

Supplement: Figure 4-1 — HFS could enhance the connectivity of other afferent inputs besides the callosal afferent in a non-specific manner. (A) Two electrodes were inserted symmetrically into both hemispheres of the auditory cortex, site L and site R. Auditory stimulus was presented to both ears. HFS was applied to site R. (B) Representative fEPSP traces of auditory responses from site L (upper) or site R (lower) before (1 and 3) and after (2 and 4) HFS-R. (C) Normalized fEPSP slopes from site L (blue spot) or site R (red block) before and after HFS-R (Site L: ns, two-way RM ANOVA, p = 0.52; Site R: **, two-way RM ANOVA, p < 0.01; N = 12). Download Figure 4-1, TIF file. [file eneuro-12-ENEURO.0045-24.2024-s004.tif]
